# Dual-SVF: a robust knowledge-guided multimodal method for structural variant filtering in long-read sequencing

**DOI:** 10.1093/bib/bbag448

**Published:** 2026-08-24

**Authors:** Chunxiao Lai, Haohao Zhang, Chong Cheng, Jing Peng, Xiaohui Yuan

**Affiliations:** Sanya Science and Education Innovation Park, Wuhan University of Technology, Building 9, Yonyou Industrial Park, Yazhou Bay Science and Technology City, Sanya, Hainan 572024, China; School of Computer Science and Artificial Intelligence, Wuhan University of Technology, No. 21 Gongda Road, Hongshan District, Wuhan, Hubei 430070, China; School of Computer Science and Artificial Intelligence, Wuhan University of Technology, No. 21 Gongda Road, Hongshan District, Wuhan, Hubei 430070, China; Wuhan Second Ship Design and Research Institute, No. 450 Zhongshan Road, Wuchang District, Wuhan, Hubei 430064, China; Sanya Science and Education Innovation Park, Wuhan University of Technology, Building 9, Yonyou Industrial Park, Yazhou Bay Science and Technology City, Sanya, Hainan 572024, China; School of Computer Science and Artificial Intelligence, Wuhan University of Technology, No. 21 Gongda Road, Hongshan District, Wuhan, Hubei 430070, China; School of Computer Science and Artificial Intelligence, Wuhan University of Technology, No. 21 Gongda Road, Hongshan District, Wuhan, Hubei 430070, China; Engineering Research Centre of Chinese Ministry of Education for Edible and Medicinal Fungi, Jilin Agricultural University, No. 2888 Xincheng Street, Jingyue District, Changchun, Jilin 130118, China; Yazhouwan National Laboratory, No. 8 Huanjin Road, Yazhou Bay Science and Technology City, Sanya, Hainan 572024, China

**Keywords:** structural variant filtering, multimodal learning, confidence-gated cross-attention mechanism, genomic prior knowledge

## Abstract

Structural variants (SVs) are key drivers of genomic diversity and disease, yet their accurate detection from long-read sequencing remains challenged by high false-positive rates caused by sequencing errors and alignment artifacts. Current filtering approaches predominantly rely on alignment structure, often overlooking sequence content and genomic context knowledge, which undermines the robustness of SV detection. To address these issues, we present Dual-SVF, a robust knowledge-guided multimodal method for SV filtering that jointly models genomic semantics (from raw sequence content) and syntactics (from sequence alignment topology). Dual-SVF integrates genomic prior knowledge, including sequence entropy, GC content, and mapping quality, through a confidence-gated cross-attention mechanism that dynamically weights modality reliability and enables mutual error correction. Validation across diverse sequencing platforms and multiple species demonstrates that Dual-SVF consistently achieves superior performance compared with state-of-the-art methods. Dual-SVF is an open-source, VCF-compatible tool, which seamlessly complements existing pipelines to ensure reliable SV filtering across noisy genomic data.

## Introduction

Structural variants (SVs) are closely associated with various genetic diseases [1]. For instance, oncogenic Alu insertions [2] and complex rearrangements like 22q11.2 deletions [3] are critical for understanding disease mechanisms and informing clinical diagnostics. Represented by Oxford Nanopore [4] and PacBio [5], long-read sequencing (LRS) overcomes repetitive elements and complex breakpoints that obstruct short-read technologies, conferring distinct advantages for large-scale SV detection in repetitive or otherwise intractable genomic regions [6]. Algorithms developed for LRS data, such as SVIM [7], cuteSV [8], Sniffles [9], and SVHunter [10], have made substantial progress in detecting SVs in the human genome. Despite its throughput, LRS suffers from 5% to 15% error rates and systematic biases, yielding high false discovery rates [11]. These artifacts, stemming from sequencing noise and low-complexity regions, necessitate an automated post-processing framework to refine SV candidates for high-confidence downstream analysis.

Existing SV filtering strategies have transitioned from labor-intensive manual inspection using visualization tools like IGV [12] and svviz [13] to integrative ensemble methods such as NextSV [14] and CombiSV [15]. While these approaches improve precision by aggregating multiple algorithms, they often incur significant computational overhead and require complex parameter coordination. Recently, deep learning has emerged as a promising paradigm for automated SV calling and filtering [16]. This development traces back to short-read sequencing, where tools like DeepSV [17] and DeepSVFilter [18] first demonstrated the efficacy of encoding alignment signatures (e.g. read depth and split reads) into images for convolutional neural network (CNN)-based classification. Subsequent work such as TensorSV [19] introduced tensor-based representations and variable topology networks to extend detection to complex types like inversions.

With the development of LRS, this paradigm has been further extended to address higher error rates and more complex genomic landscapes [20]. Frameworks such as SVision [21] and CSV-Filter [22] leverage CNNs to recognize topological signatures from alignment operations (e.g. CIGAR strings), while SVHunter [10] combines CNNs with Transformers to model both local features and long-range global dependencies. More recently, methods like DASV [23] have explored dual-attention mechanisms to detect deletions. However, these “syntactic-only” approaches are inherently limited by their reliance on alignment artifacts, which are prone to distortion in repetitive or low complexity regions. Such sensitivity to sequencing errors often leads to misclassification without complementary semantic support.

A critical, yet often overlooked, dimension is the raw nucleotide-level sequence content. Nucleotide sequences carry rich “semantic” information [24], including sequence complexity, GC content, and repeat motifs, which provides a biologically meaningful signal independent of alignment syntax [25]. Multi-modality learning offers a principled framework for integrating such heterogeneous biological signals [26, 27]. By aligning and fusing features from distinct modalities, models can achieve greater interpretability and robustness, a principle successfully applied in multi-omics data integration [28]. Furthermore, the integration of genomic prior knowledge (GPK) [29] (e.g. mapping quality and sequence entropy) is vital for guiding models to distinguish a true SV from sequencing artifacts, ensuring that the learned representations remain biologically consistent.

To overcome these barriers, we develop Dual-SVF, an advanced computational method that redefines SV filtering through a dual-perspective feature integration strategy. Unlike traditional filters that treat alignment data as a monolithic input, Dual-SVF models genomic information through two complementary streams: a semantic stream capturing the intrinsic patterns of raw nucleotide sequences and a syntactic stream extracting the spatial topology of read alignments. Central to our architecture is the Semantic–Syntactic Mutual Correction Mechanism (SSMCM). This module employs confidence-gated components to adaptively reconcile discrepancies between modalities, effectively suppressing noise in regions where alignment quality is poor or sequence complexity is high. Furthermore, Dual-SVF ensures that filtered candidates align with known genomic characteristics by incorporating domain-specific biological constraints, such as sequence entropy and local GC content, directly into the learning process. Benchmarking results across multiple species and diverse LRS platforms demonstrate the superior precision and robust cross-species generalizability of our method. As an open-source, VCF-compatible tool, Dual-SVF offers a reliable solution to enhance the accuracy of structural variation discovery in large-scale genomic studies.

## Materials and methods

**Figure 1:**
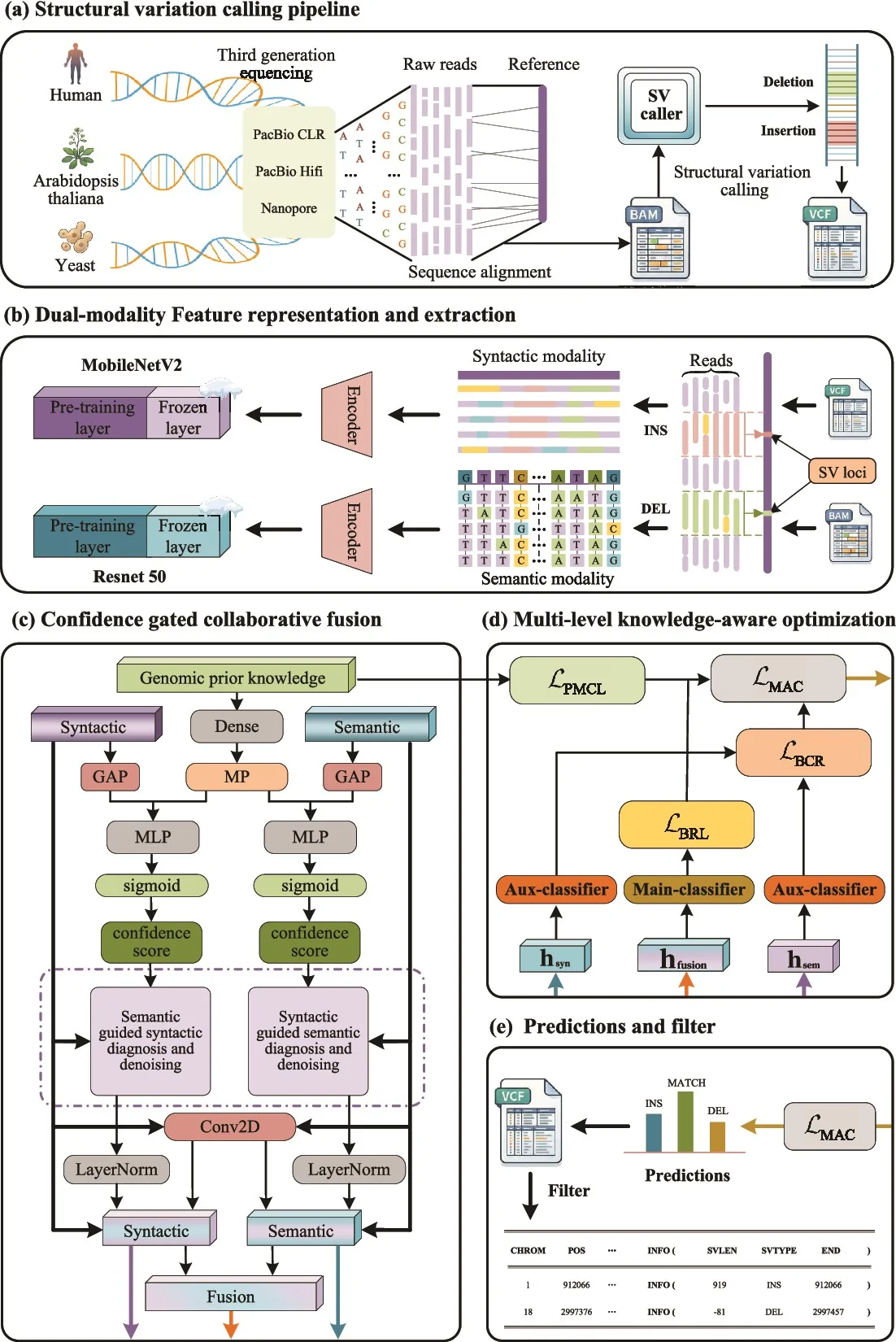
The overall workflow of Dual-SVF method; the pipeline comprises four main steps to filter SVs. (a) Initial SV calling pipeline across multiple species and platforms. (b) Dual-modality feature extraction using decoupled ResNet-50 and MobileNetV2 encoders for semantic (sequence) and syntactic (alignment) modalities. (c) The SSMCM, which reconciles modalities via confidence-gated fusion guided by genomic priors (e.g. GC content and entropy). (d) Multi-task training with a joint loss function ($\mathcal{L}_{\textrm{BRL}}$, $\mathcal{L}_{\textrm{BCR}}$, $\mathcal{L}_{\textrm{PMCL}}$, and $\mathcal{L}_{\textrm{MAC}}$) to ensure cross-modal consistency. (e) SV prediction and automated filtering to produce high-confidence VCF results.

Dual-SVF is a knowledge-guided multimodal method for high-confidence SV filtering in LRS data. As illustrated in Fig. 1, the workflow consists of four streamlined stages:

(i) Feature representation and extraction: Following initial SV caller, Dual-SVF extracts semantic features from raw sequences and syntactic features from alignment topologies. These are processed through decoupled ResNet-50 [30] and MobileNetV2 [31] encoders to generate high-level representations.
(ii) Confidence-gated collaborative fusion: To reconcile modality discrepancies, the SSMCM utilizes a confidence-gated module. By integrating genomic priors, it adaptively weights modality reliability and suppresses noise in complex genomic regions.
(iii) Multi-level knowledge-aware optimization: The method is optimized via multi-task learning, employing a joint loss function $\mathcal{L}_{\textrm{MAC}}$ to ensure cross-modal consistency and enforce biological constraints during training.
(iv) Predictions and filter: Dual-SVF generates type-specific SV predictions to filter false positives from the input set, ultimately producing a refined, standardized VCF file for reliable downstream analysis.

### Feature representation and extraction

The Dual-SVF pipeline begins by extracting targeted genomic windows around candidate SV loci. As illustrated in Fig. 1a, initial SV candidates are identified by aligning long reads to a reference genome and processing them through standard callers such as cuteSV or Sniffles. Dual-SVF isolates candidate SV loci by extracting targeted genomic windows centered at predicted coordinates from initial callers like cuteSV or Sniffles. This localized approach reduces computational overhead while focusing on specific variation signals. For each locus, we extract two modalities to capture distinct genomic signatures (Fig. 1b).

**Syntactic modality (alignment topology):** This stream encodes the logic of CIGAR strings (e.g. matches, indels, andsoft clips) into a spatial representation. Given the structured nature of alignment signals, we employ a lightweight MobileNetV2 encoder. This choice ensures high computational efficiency while maintaining the capacity to extract robust topological patterns.

**Semantic modality (sequence content):** To resolve alignment ambiguities in complex regions, this stream processes raw nucleotide sequences surrounding breakpoints. We utilize a ResNet-50 encoder to capture intricate biological motifs, such as sequence entropy and repeat motifs. The deeper residual structure is specifically leveraged to model complex long-range dependencies that shallower networks often miss.

### Confidence-gated collaborative fusion

To synergistically integrate decoupled features, Dual-SVF employs the SSMCM (Fig. 1c). This mechanism transcends simple concatenation by adaptively reconciling discrepancies between modalities guided by genomic priors, following a “diagnose-then-Rectify” logic (detailed in Supplementary Fig. S1).

**Knowledge-informed latent embedding:** Dual-SVF incorporates explicit biological priors (e.g. sequence entropy, GC content), which are mapped to a latent context denoted as a prior vector $\mathbf{P}$ (Supplementary Table S6). This vector is transformed into a latent context $\mathbf{z}_{p}$ via a dense layer, serving as a biologically grounded anchor for cross-modal assessment. This architecture ensures that the multimodal fusion remains consistent with the physicochemical properties of the genomic region rather than relying on purely data-driven statistical correlations. The decoupled contributions of these heterogeneous priors and the underlying mechanistic pathways of prior-guided feature learning have been extensively validated through ablation and interpretive analysis (detailed in Supplementary Section 3.4).

**Confidence-gated modality weighting:** To address the heterogeneity of different modalities, a confidence-scoring module evaluates the reliability of the semantic ($\mathbf{h}_{\textrm{sem}}$) and syntactic ($\mathbf{h}_{\textrm{ syn}}$) streams. The confidence scores *s* are computed as: 


(1) \begin{align*}& s_{\textrm{sem}} = \sigma\bigl(f(\mathbf{h}_{\textrm{sem}}, \mathbf{z}_{p})\bigr), \quad s_{\textrm{syn}} = \sigma\bigl(f(\mathbf{h}_{\textrm{syn}}, \mathbf{z}_{p})\bigr)\end{align*}

where *σ * is the sigmoid function, and *f(· )* represents a learnable mapping. These scores act as dynamic filters; for instance, in repeat-rich loci where alignment is distorted (low $s_{\textrm{ syn}}$), the method autonomously upweights the semantic stream. This adaptive prioritization mimics the logic of expert manual curation, where sequence motifs are leveraged to verify ambiguous alignments.

**Mutual diagnosis and feature denoising:** The features undergo a bidirectional “diagnosis” process to filter out platform-specific artifacts. By utilizing a gated cross-attention logic, the semantic stream provides a reference for denoising syntactic signals, while the syntactic stream grounds semantic patterns within a topological context. The final integrated representation $\mathbf{H}_{\textrm{fusion}}$ is computed as: 


(2) \begin{align*}& \mathbf{H}_{\textrm{fusion}} = \mathrm{Conv2D}\bigl([s_{\textrm{sem}} \cdot \mathbf{h}_{\textrm{sem}} \oplus s_{\textrm{syn}} \cdot \mathbf{h}_{\textrm{syn}}]\bigr)\end{align*}

This synergistic fusion ensures that Dual-SVF maintains high fidelity across diverse genomic landscapes, effectively bridging the gap between raw sequence semantics and alignment syntactics.

### Multi-level knowledge-aware optimization

To mitigate modality conflicts and ensure biological consistency, Dual-SVF employs a multi-level optimization strategy. As shown in Fig. 1d, we systematically inject genomic priors into the feature, prediction, and output spaces to suppress heterogeneous noise.

**Balanced representation learning (BRL):** Dual-SVF utilizes a joint architecture **$\mathcal{L}_{\textrm{BRL}}$** with a primary classifier and two auxiliary heads. This design resolves the “modality dominance” issue by forcing each stream to extract independent and discriminative patterns before fusion, ensuring a balanced contribution to the final SV classification.

**Prior-modulated contrastive loss (PMCL):** Standard contrastive learning often fails in repetitive regions due to inherent distribution shifts. We introduce PMCL to calibrate feature alignment using GPK. We compute a prior-based similarity weight $w_{ij}$ to modulate the contrastive objective: 


(3) \begin{align*}& w_{ij} = \exp\left(-\frac{\|\mathbf{p}_{i} - \mathbf{p}_{j}\|_{2}^{2}}{2\sigma^{2}}\right)\end{align*}

By integrating GPK as a spatial navigator, PMCL forces the model to cluster samples with similar biological backgrounds even when raw sequencing signals are noisy.

**Biological consistency regularization (BCR):** To enforce logical coherence, BCR penalizes cross-modal contradictions based on the bioinformatic prior that true SVs should manifest consistent signals across both alignment and sequence contexts. We minimize the Jensen–Shannon (JS) divergence between unimodal predictions to ensure this consistency.

The framework is optimized end-to-end using the multi-level adaptive constraint (MAC) loss: 


(4) \begin{align*}& \mathcal{L}_{\textrm{MAC}} = \mathcal{L}_{\textrm{BRL}} + \alpha \mathcal{L}_{\textrm{PMCL}} + \beta \mathcal{L}_{\textrm{BCR}}\end{align*}

This tri-level supervision provides context-awareness, structural consistency, and discriminative power, enabling robust SV filtering across challenging landscapes (see Supplementary Section 3.2).

### Predictions and filter

The final stage of the Dual-SVF pipeline transforms the optimized joint representations into high-fidelity variant calls through a coordinated inference and filtering process (detailed in Fig. 1e).

**Prior-aware dynamic prediction:** To achieve high-precision refinement, Dual-SVF employs a dynamic filtering mechanism. Unlike traditional filters with static thresholds, our decision boundary is adaptively calibrated by the learned genomic priors. This allows the model to autonomously increase filtering stringency in complex genomic regions (e.g. repeat-rich loci), effectively suppressing sequencing artifacts that often confound traditional heuristic rules.

**Probabilistic classification:** During inference, the primary classifier utilizes the fused representation $\mathbf{h}_{\textrm{fusion}}$ to generate a posterior probability distribution across three categories: noise (NEG), insertions (INS), and deletions (DEL). A candidate locus is assigned the type with the maximum probability: $\hat{y} = \arg \max (\mathbf{p}_{\textrm{predict}})$.

This integrated strategy ensures that each variant call is substantiated by evidence from both alignment topologies and sequence semantics. The final output is a refined, standard VCF file, ready for high-confidence downstream biological analysis.

## Results

### Training sample generation

In this study, INS and DEL recorded in the VCF file are defined as positive samples. However, standard VCF records inherently lack negative instances, which are essential for robust model training. To address this limitation, we develop the *Non-overlapping Genomic Intervals Random Bucket Sampling* (NIRBS) algorithm (detailed in Supplementary Section 3.3) to generate high-fidelity negative instances. Prior to sampling, a chromosome-wide statistical analysis reveals that the frequency and length of SV types approximately follow a Poisson-like distribution across diverse genomic landscapes. Utilizing this identified distribution as a generative prior, the NIRBS algorithm iteratively extracts nonoverlapping genomic intervals from the reference genome. The bucket sampling approach ensures that the length distribution of generated negatives closely mimics that of the positive samples, thereby preventing the model from over-relying on sequence length as a classification shortcut. Downstream spatial profiling of these sampled intervals (detailed in Supplementary Sections 3.3.3 and 3.3.4) confirms the empirical validity of this strategy, demonstrating that the generated negatives naturally encapsulate complex biological noise from alignment-deceptive regions (such as centromeres and repetitive elements) rather than simplified uniform backgrounds. This strategy maintains both distributional consistency and genomic independence, providing high-fidelity negative instances that facilitate the learning of nuanced biological features and resolve realistic performance boundaries.

### Benchmarking on simulated data across multiple species

To validate the robustness of Dual-SVF in SV filtering, we conduct extensive benchmarking using diverse simulated datasets. We employ *SURVIVOR* [32] and *PBSIM2* [33] to generate LRS data for three phylogenetically distinct species with varying genomic complexities: *Homo sapiens* (3.0 Gb, 45%–50% repeats), *Arabidopsis thaliana* (117 Mb, 27% repeats), and *Saccharomyces cerevisiae* (12.0 Mb, <4% repeats) are chosen to represent complex, model plant, and streamlined microbial genomes, respectively. Both Oxford Nanopore Technologies (ONT) and PacBio Continuous Long Read (CLR) datasets are generated for each species to capture platform-specific noise profiles, including the stochastic error rates of CLR and the systematic biases of ONT. The simulated reads are aligned via *minimap2*, and candidate variants are identified using three state-of-the-art (SOTA) callers: *cuteSV*, *Sniffles*, and *SVIM*. Dual-SVF is then applied to refine these candidates. Finally, *SURVIVOR* is utilized to evaluate the consistency between VCF records before and after filtering, demonstrating the method’s universal capability to discern true biological variants from diverse genomic backgrounds.

**Figure 2:**
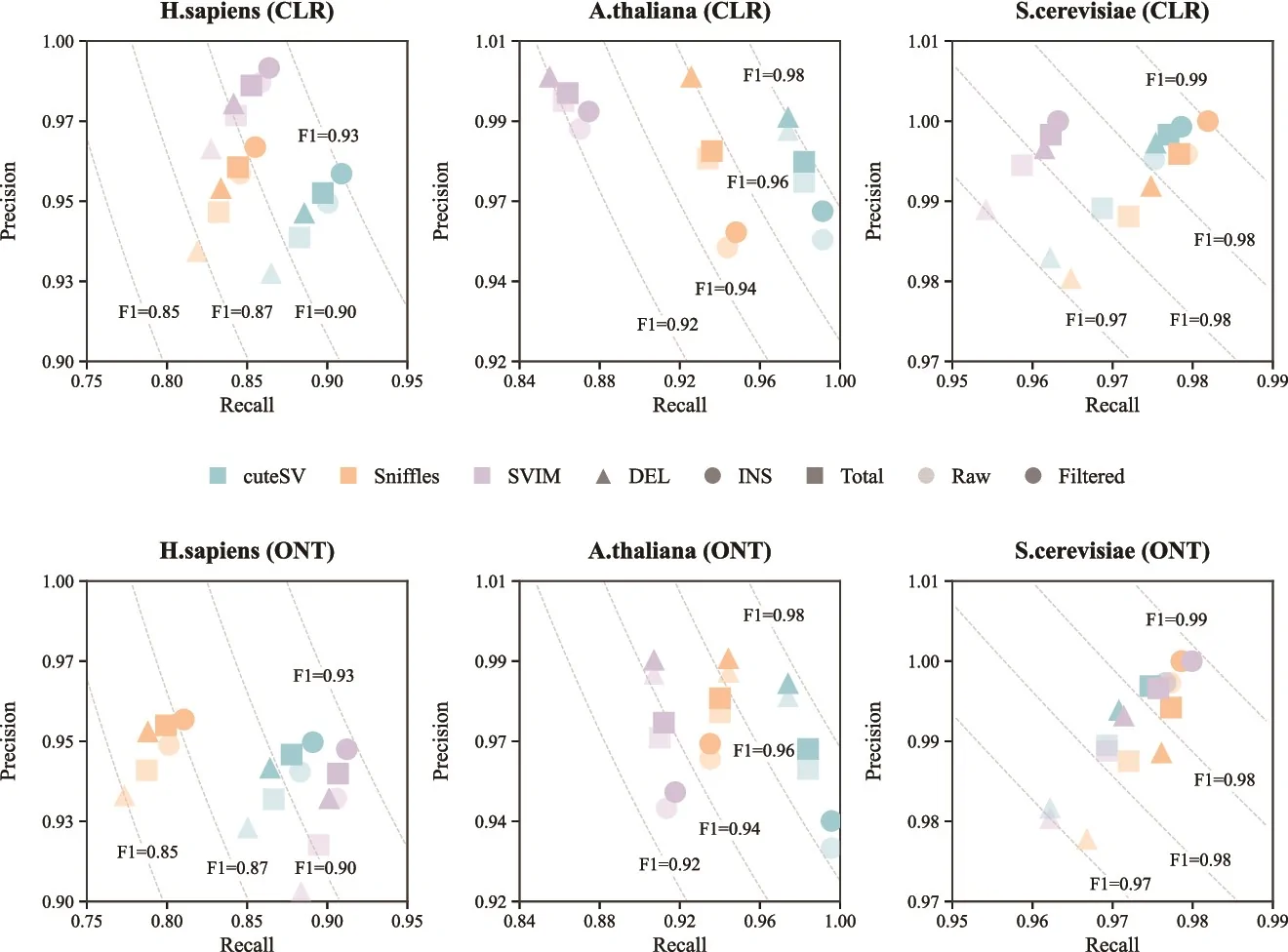
Performance of SV filtering across multiple species and platforms at 30*× * coverage. Precision-recall curves illustrate results for *H. sapiens*, *A. thaliana*, and *S. cerevisiae*. The top and bottom rows represent PacBio CLR and ONT data, respectively. Light-colored markers denote raw calls from cuteSV, Sniffles, and SVIM, while dark-colored markers represent corresponding results after Dual-SVF filtering. Isoparametric lines indicate F1-score. Sub-panels categorize performance into total variants, INS, and DEL.

At a sequencing depth of 30*× *, Dual-SVF exhibits superior cross-species and cross-platform performance. As shown in Fig. 2, the refined call sets consistently shift the precision-recall curves of all baseline tools toward the upper-right quadrant. In the complex human genome, Dual-SVF achieves a robust F1-score of 0.94 on both ONT and CLR platforms, while in more compact genomes, peak F1-scores reach 0.98 in *A. thaliana* and 0.99 in *S. cerevisiae*. The model maintains high fidelity for both INS and DEL, suggesting that the integration of semantic sequence streams and syntactic alignment flows effectively captures invariant structural features across divergent genomic contexts.

To evaluate operational stability under resource-constrained conditions, we analyze performance across a gradient of sequencing depths (5*× *, 10*× *, and 30*× *; Supplementary Fig. S4). In ultra-low coverage scenarios (5*× *), where physical evidence is sparse and sequencing noise is prevalent, Dual-SVF maintains a substantial lead over raw detection methods. For example, in the 5*× * human ONT dataset, Dual-SVF recalibrates the classification boundary to achieve an F1-score of 0.76, whereas baseline tools suffer from significantly higher false-discovery rates. This performance gain establishes the theoretical efficacy of our MAC-calibrated filtering, demonstrating that the model effectively bridges the gap left by sparse physical evidence by leveraging learned multimodal correlations. As coverage increases, Dual-SVF progressively exploits additional evidence to further refine SV calls.

Beyond coverage gradients, the structural complexity of noncanonical variants introduces distinct topological challenges. Extended evaluation on advanced structural architectures reveals that while Dual-SVF reliably refines Duplications (DUP), its performance gains remain constrained for Inversions (INV) and Translocations (TRS). Due to the localized receptive field of the current dual-stream encoders, the framework excels at parsing spatially contiguous indel footprints but experiences limitations when resolving distal breakpoint associations or orientation-sensitive strand cues. This honest delineation of architectural boundaries highlights the baseline constraints of localized feature extraction in complex genomic rearrangements (detailed in Supplementary Section 4.3).

In conclusion, these benchmarks confirm that Dual-SVF represents a robust and generalizable refinement approach capable of significantly enhancing SV filtering accuracy across diverse evolutionary lineages and baseline error models. While this superior performance demonstrates the synergistic efficacy of coupled semantic and syntactic representation learning, our evaluation also delineates its structural boundary: the current architecture’s reliance on localized feature extraction inevitably leaves a performance gap when resolving massive, noncontiguous rearrangements such as translocations. Recognizing this boundary, future work will focus on integrating global sequence modeling techniques and expansive receptive fields to better accommodate complex, multi-breakpoint rearrangements. Nevertheless, the current framework’s capacity to autonomously calibrate filtering stringency and mitigate biological noise establishes it as a highly reliable component for high-fidelity SV calling.

### Benchmarking across multi-platform and coverage gradients on HG002

To comprehensively assess the filtering efficacy of Dual-SVF on the real-world HG002 human benchmark, we analyze three platforms with distinct error profiles: PacBio CLR (stochastic errors), Oxford Nanopore (ONT, systematic biases), and PacBio CCS (high-fidelity HiFi reads). Using *samtools*, we perform random downsampling to establish coverage-gradient datasets. Specifically, the evaluation encompasses PacBio CLR (69*× *  *→ * 35*× *  *→ * 20*× *  *→ * 10*× *  *→ * 5*× *), ONT (48*× *  *→ * 20*× *  *→ * 10*× *  *→ * 5*× *), and CCS (28*× *  *→ * 10*× *  *→ * 5*× *). This experimental design aims to demonstrate the method’s robustness in complex human genomic environments across various data qualities. Candidate SVs detected by *cuteSV*, *Sniffles*, and *SVIM* are subsequently refined by Dual-SVF to evaluate the consistent performance gains across diverse sequencing contexts.

**Figure 3:**
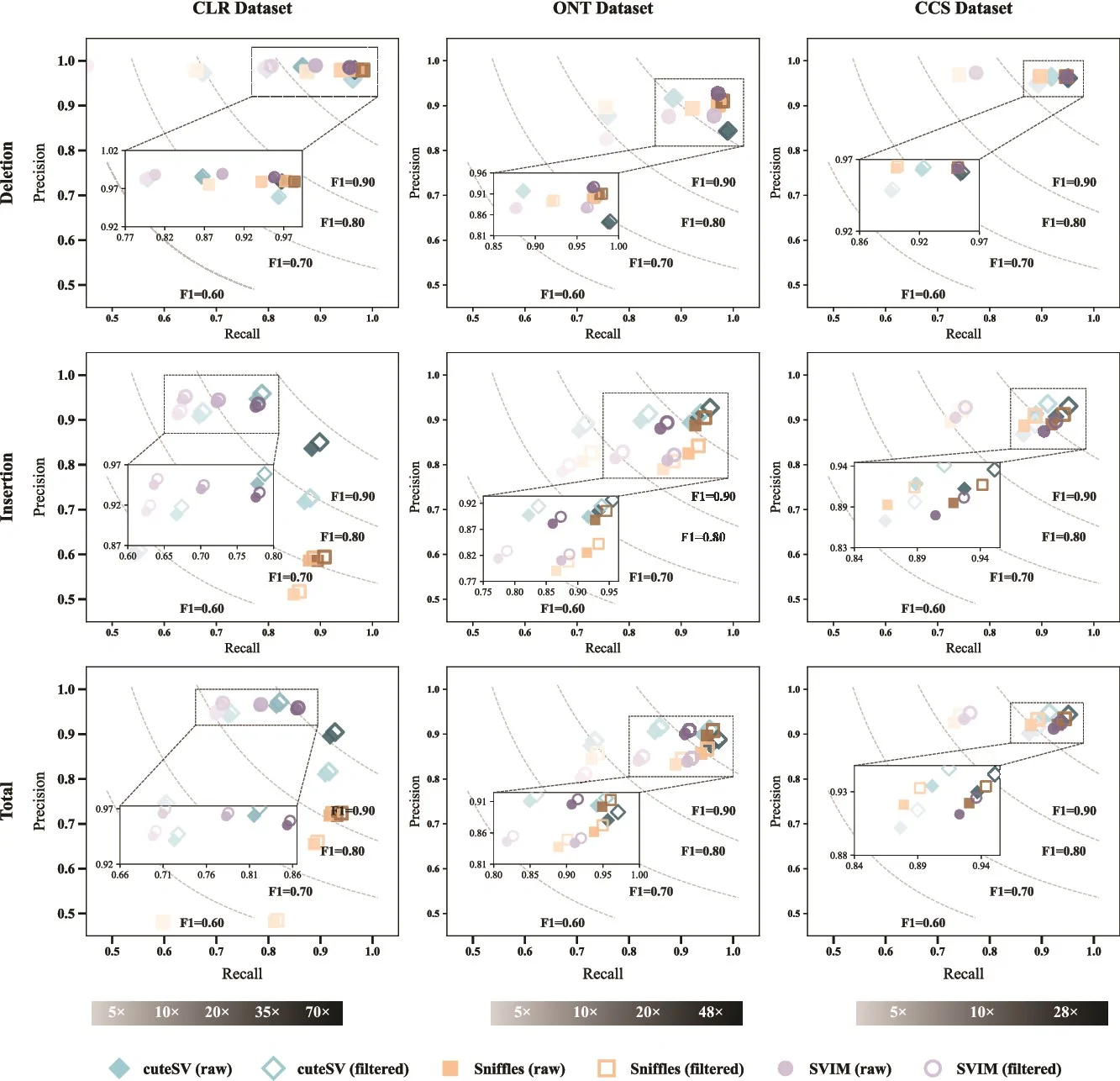
SV filtering performance of Dual-SVF on HG002 across multiple platforms and coverages. Panels are categorized by variant types (top: DEL; middle: INS; bottom: ALL) and sequencing platforms (left: CLR; middle: ONT; right: CCS). Distinct marker shapes represent three baseline detectors (diamonds: cuteSV; squares: Sniffles; circles: SVIM), where solid markers denote raw calls and hollow markers denote results after Dual-SVF filtering. Marker shading intensity indicates sequencing depth, with darker shades representing higher coverage. Isoparametric lines indicate F1-score.

As illustrated in Fig. 3, Dual-SVF exhibits superior filtering efficacy across all three real-world platforms. For the high-fidelity CCS dataset, the model achieves near-perfect precision while maintaining exceptional recall. In the noise-heavy CLR and ONT datasets, the refinement is even more pronounced. Notably, the model demonstrates a consistent performance advantage in INS compared with DEL. In these authentic biological contexts, the semantic stream proves particularly vital, as it decodes the explicit nucleotide content of inserted segments to provide more discriminative signals than the gap-based syntactic features utilized in deletion events.

The robustness of Dual-SVF to sequencing depth is further reinforced under the nonrandom noise profiles of the HG002 benchmark. At ultra-low coverage (5*× *), the model effectively purges artifactual candidates that typically overwhelm traditional callers in practice. While earlier simulations established the model’s fundamental reliability, these HG002 results confirm its practical resilience against the systematic biases and library preparation artifacts inherent in human genomic data. As depth increases, Dual-SVF capitalizes on the higher-density signal to reach near-saturation in precision, validating its adaptability to the heterogeneous noise of diverse platforms. Given the inherent reference gaps in GRCh37, we next extend our validation to the gapless T2T-CHM13 genome to assess the model’s ability to discriminate true variants from reference-induced artifacts.

### Specificity evaluation on the T2T-CHM13 genome

To comprehensively evaluate SV detection and filtering performance across diverse genomic contexts, we implement a dual-reference benchmarking strategy utilizing the CHM13 sample. By aligning CHM13 long-read data to the gap-heavy GRCh38 reference, we employ assembly-based variants as the ground truth to evaluate the retention of genuine SVs. Conversely, aligning the same reads to the gapless T2T-CHM13 genome, which serves as a nonchimeric and high-quality haploid reference, allows us to quantify the model’s capacity to suppress technical artifacts. Under this self-reference setting, an ideal tool theoretically detects zero variants.

**Figure 4:**
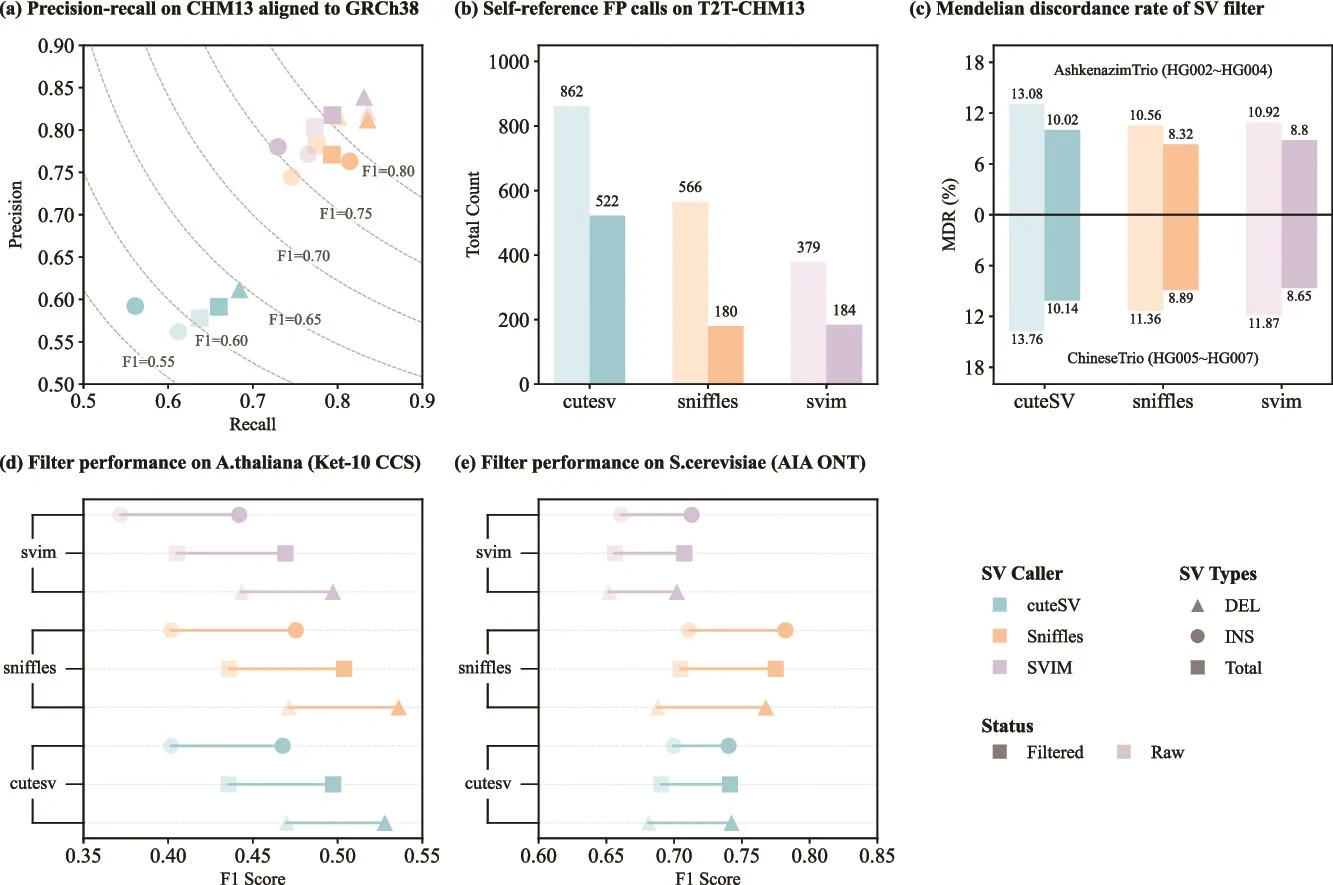
Technical specificity and biological consistency evaluation. (a) Precision-recall curves of SV filtering on CHM13 reads aligned to GRCh38, where darker markers represent Dual-SVF filtered results. (b) Comparison of false-positive (FP) call counts on the T2T-CHM13 self-alignment benchmark, demonstrating the reduction in artifactual variants. (c) Mendelian Discordance Rate (MDR) across Ashkenazim and Chinese trios, highlighting the improved biological integrity after filtering.

Initially, we assess precision, recall, and F1-score by comparing alignment-based calls against the Dipcall-generated benchmark set [34] on the GRCh38 reference. As illustrated in Fig. 4a, the application of our deep learning-based filter significantly improves precision across all tested tools, including *cuteSV*, *Sniffles*, and *SVIM*. The filtered results, indicated by darker symbols, demonstrate a substantial shift toward the upper-right quadrant of the precision-recall space, maintaining high recall while effectively pruning false-positive calls. This performance confirms that Dual-SVF is highly effective at capturing a high proportion of genuine SVs while distinguishing them from the technical noise frequently encountered in the complex repetitive regions of the GRCh38 genome.

We subsequently perform a self-alignment to the T2T-CHM13 genome [35] to evaluate resistance against false positives. As shown in Fig. 4b, raw detection tools identify a considerable number of artifactual variants, with *cuteSV*, *Sniffles*, and *SVIM* yielding 862, 566, and 379 false-positive calls, respectively. Our method successfully mitigates these artifacts, reducing the counts to 522, 180, and 184, respectively. This substantial reduction highlights the model’s superior capability to filter out reference-induced artifacts and technical noise that often confound traditional algorithms.

While these CHM13 benchmarks confirm technical specificity and noise suppression in a variant-free context, a comprehensive evaluation must also ensure that the filtered results conform to fundamental biological laws. Consequently, we extend our validation to assess Mendelian inheritance consistency within standard human trio pedigrees, thereby confirming the biological authenticity of the preserved SV signals.

### Mendelian consistency in human trio family

While the preceding benchmarks on HG002 and T2T-CHM13 confirm technical precision and specificity, evaluating inheritance fidelity from parents to offspring provides a more stringent assessment of biological authenticity. To validate that the filtered results conform to fundamental Mendelian principles, we extend our validation from single-sample benchmarks to Mendelian inheritance consistency within standard human trio pedigrees. We employ the MDR [36] to quantify the consistency of Dual-SVF in filtering variants within parent–offspring samples.

As illustrated in Supplementary Tables S14 and S15, and Figs S5 and S6, we assess the MDR across two diverse genomic backgrounds including the Ashkenazim Trio (HG002–HG004) and the Chinese Trio (HG005–HG007). Across all tested SV detection tools, such as *cuteSV*, *Sniffles*, and *SVIM*, the application of the Dual-SVF method results in a consistent and significant reduction in the MDR. This improvement is clearly indicated in Fig. 4c by the darker bars representing filtered results compared to the lighter bars of the unfiltered calls. For instance, in the Ashkenazim Trio, the MDR for *Sniffles* drops from 10.56% to 8.32%, while in the Chinese Trio, the MDR of *SVIM* improves from 11.87% to 8.65%. These results confirm that the method maintains high biological integrity by effectively identifying and removing false-positive variants that violate inheritance patterns.

Mendelian inheritance analysis confirms that Dual-SVF maintains high biological integrity by effectively segregating heritable SVs from stochastic noise in the human genome. However, a robust SV filtering method must transcend human-specific genomic patterns. To demonstrate universal applicability, we shift our validation from human pedigrees to real-world datasets of *A. thaliana* and *S. cerevisiae*, benchmarking the model against the vastly different genomic architectures of plants and fungi.

### Cross-species robustness and practical utility

To evaluate the cross-species robustness and practical utility of Dual-SVF beyond the human lineage, we extend our validation to real long-read datasets of *A. thaliana* [37] and *S. cerevisiae* [38]. Unlike simulated data, these real-world samples encompass complex biological noise and species-specific structural variations that challenge the generalization limits of deep-learning models. By applying our method to the compact genome of *A. thaliana* and the highly streamlined genome of *S. cerevisiae*, each represented by two independent sequencing datasets, we aim to demonstrate the model’s capacity to transcend specific genomic architectures and maintain high-fidelity SV refinement across diverse sequencing platforms.

As illustrated in Fig. 4d and e, and Supplementary Fig. S7, Dual-SVF exhibits remarkable adaptability across these evolutionarily distant species. In the analysis of *A. thaliana* Ket-10 (Fig. 4d), which utilizes PacBio CCS data, the filtered results show a consistent and significant increase in F1-score across all three underlying callers compared to their raw outputs. A similar trend is observed in the *S. cerevisiae* AIA dataset (Fig. 4e), where the method effectively refines SV calls from ONT sequencing data.

Although the absolute F1-score in these species are lower than those observed in human benchmarks, this discrepancy primarily stems from the original design of these datasets. Specifically, since the data are primarily curated for graph-based pangenome analysis, focusing on population-level genomic diversity rather than high-precision single-sample SV detection, the baseline noise profiles differ from standard benchmark sets. Despite these inherent dataset limitations, the relative gain in performance remains substantial. Nevertheless, as further detailed in Supplementary Fig. S7, the universal improvement in F1-score across different biological kingdoms confirms that Dual-SVF is not overfitted to human-specific patterns. Instead, it possesses the robust generalization capabilities required for diverse genomic research, proving its value as a generalized tool for high-precision structural variation analysis.

Finally, to decouple the specific architectural benefits of our multimodal design from generic data-reduction effects, we compared Dual-SVF’s filtering efficacy against the SOTA single-modality filter, CSV-Filter. As summarized in Supplementary Table S13 (and further detailed in Supplementary Section 4.6), univariate filters operating purely on alignment syntax (CIGAR) frequently compromise Precision due to topological distortions in repeat-rich regions. Conversely, Dual-SVF leverages raw sequence content as an independent biological reference frame to dynamically cross-verify and rectify these ambiguous alignments. This cross-modal synergy delivers consistent, peak F1-score gains across divergent platforms and baseline callers, establishing the empirical superiority and universal utility of our joint framework.

### Multimodal fusion and ablation analysis

To rigorously evaluate the contributions of individual architectural components, we benchmarked diverse backbone architectures; as revealed in the Supplementary 6.2 section, there is a distinct divergence in modality-specific requirements. Subsequently, we conducted an extensive ablation study to evaluate single-modality versus dual-modality configurations. Dual-SVF integrates a *Syntactic* modality, which captures local structural topology from alignment signals, with a *Semantic* modality that extracts intrinsic biological features directly from raw nucleotide sequences. This analysis quantifies the supplementary value of sequence-level information in refining syntactic features, demonstrating the necessity of multidimensional feature integration for complex genomic analysis.

**Figure 5:**
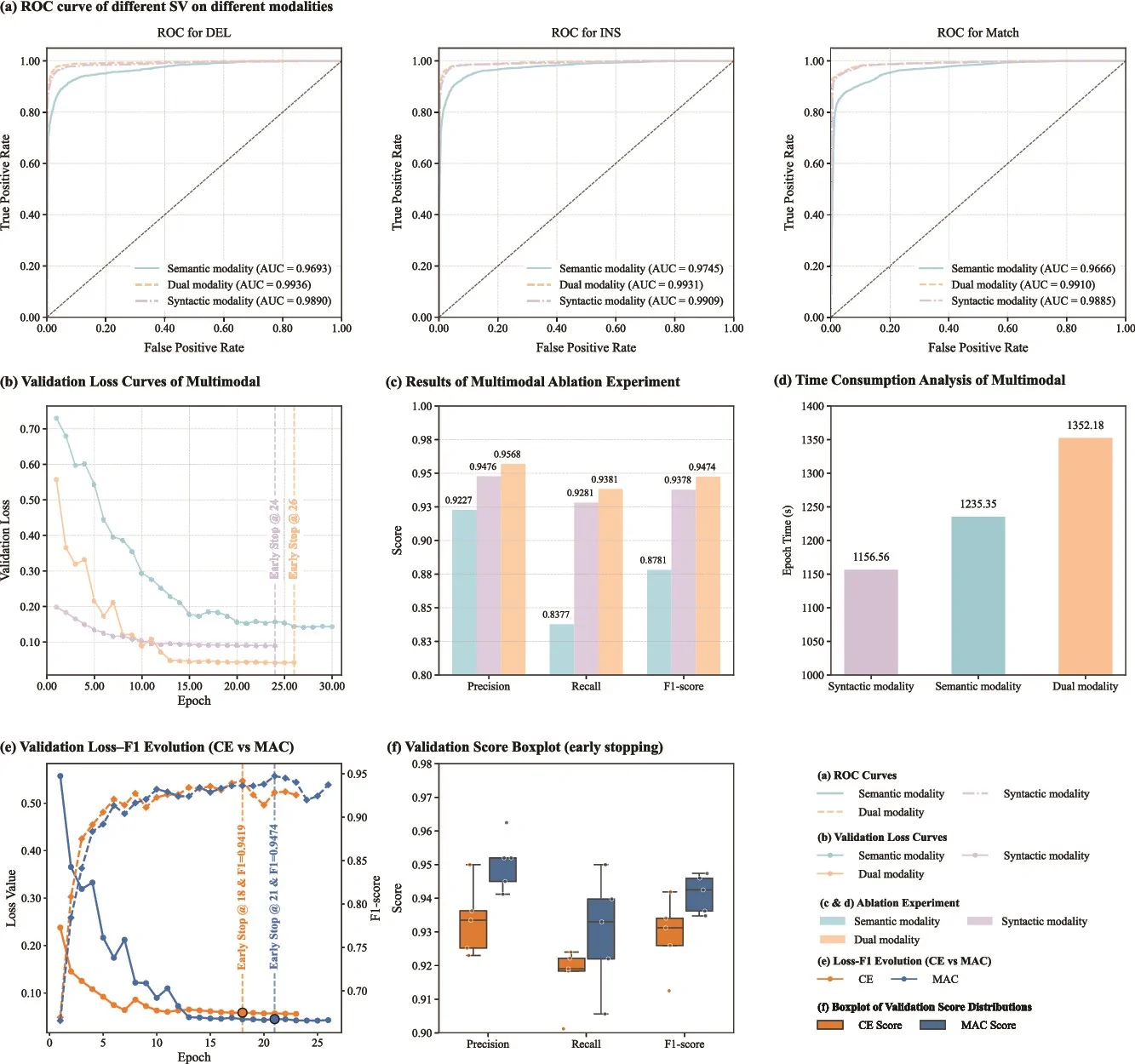
Ablation study and performance evaluation of the Dual-SVF architecture. (a) ROC curves and AUC values for DEL, INS, and noise (NEG) categories. (b) Validation loss trajectories demonstrating convergence stability. (c) Quantitative performance comparison (Precision, Recall, andF1) for Syntactic, Semantic, and Dual modalities. (d) Computational efficiency analysis. (e–f) Performance stability comparison between MAC loss and standard Cross-Entropy (CE) loss.

The ROC analysis in Fig. 5a demonstrates the superior diagnostic power of the dual-modality model across all primary SV categories. For DEL, INS, and NEG, the fusion model consistently achieves the highest Area Under the Curve (AUC), reaching 0.9936, 0.9931, and 0.9910, respectively. While the syntactic modality provides a robust baseline with AUC values exceeding 0.98, the addition of semantic information drives the model toward near-perfect classification. This trend underscores that the deep features hidden within nucleotide sequences are essential for resolving inherent ambiguities in complex genomic regions where alignment-based structural signals alone are insufficient.

To uncover the underlying factors behind this statistical superiority, we further investigated the model’s capacity to resolve specific genomic failure modes that frequently confound single-modality baselines. Our mechanistic analysis reveals that the dual-modality framework effectively rectifies systematic detection errors triggered by deceptive alignment artifacts or high-density background noise. By dynamically cross-referencing sequence-level contexts with alignment topologies, Dual-SVF prevents the model from falling into localized statistical shortcuts, ensuring robust variant discrimination even within highly repetitive and low-complexity genomic landscapes (detailed in Supplementary Section 4.4).

Regarding model evolution and training performance, the validation loss curves in Fig. 5b reveal that the dual-modality model exhibits the most rapid and stable convergence trajectory, reaching significantly lower loss values than either single-modality baseline. Although the fusion of dual streams introduces a marginal increase in computational complexity, resulting in an epoch time of 1352.18 s (Fig. 5d), this overhead is a justified trade-off for the substantial gains in biological accuracy. The convergence behavior suggests that the semantic information derived from base sequences acts as a critical regularizer, guiding the dual-stream architecture toward a more robust global optimum.

A quantitative comparison in Fig. 5c highlights the foundational role of the Syntactic stream and the essential calibration provided by the Semantic modality. Independently, the syntactic stream achieves an F1-score of 0.9378 and a recall of 0.9281, outperforming the standalone semantic modality, which records an F1-score of 0.8781 and a recall of 0.8377. These results indicate that highly structured alignment operations provide robust primary anchors for canonical variations. However, the joint dual-modality configuration achieves the ultimate peak performance, reaching a top F1-score of 0.9474, demonstrating that cross-modal semantic interaction is vital to rectify localized structural distortions.

Finally, the results validate the superiority of the multi-level adaptive constraint (MAC) loss over the traditional cross-entropy (CE) baseline. As shown in Fig. 5e, the dual-modality model utilizing MAC loss reaches its optimal F1-score more rapidly and with greater stability than the CE-based version. The boxplots with explicit swarm points in Fig. 5f further confirm this optimization stability, where the MAC-driven configuration results in a systematic upward shift of the entire performance distribution, securing higher median values and markedly elevated performance floors across all independent training runs. This consistency indicates that the complementary nature of the alignment syntactics and sequence-level semantics, combined with the MAC loss, allows the method to effectively and reliably distinguish true biological variants from stochastic noise across independent training sessions.

## Discussion and conclusion

The accurate identification of SVs remains a pivotal challenge in genomic research, primarily due to the complex interplay between sequencing noise and genomic architectural diversity. In this study, we present Dual-SVF, a multimodal refinement method that synergistically integrates alignment-based syntactic features with sequence-level semantic information. Through extensive benchmarking across simulated datasets, high-confidence human benchmarks, and diverse nonhuman species, our results consistently demonstrate that Dual-SVF significantly enhances SV filtering precision and biological integrity, especially in challenging low-coverage scenarios.

A key finding of our study is the decisive role of the semantic modality in SV refinement. While traditional alignment-based tools rely heavily on geometric signals, such as read depth and split-read clusters, Dual-SVF leverages the intrinsic information embedded within the raw nucleotide sequences. As demonstrated in our ablation analysis, the semantic stream provides a foundational understanding of the genomic content, which effectively resolves ambiguities in repetitive regions where alignment signals are often confounded by stochastic noise. This dual-stream approach allows for an autonomous calibration of filtering stringency, striking a superior balance between sensitivity and specificity that transcends species-specific patterns.

Furthermore, the robustness of Dual-SVF across diverse evolutionary lineages, ranging from the streamlined genome of *S. cerevisiae* to the complex and repeat-rich landscapes of *H. sapiens* and *A. thaliana*, underscores its generalizability. The reduction in MDR across multiple human trios further validates that our method preserves true heritable variants while effectively segregating them from reference-induced artifacts and platform-specific biases. This stability suggests that Dual-SVF could serve as a reliable component in large-scale population genomic studies and clinical diagnostics where high-fidelity SV calls are paramount.

The rapid emergence of genome foundation models presents a compelling frontier for further advancing SV refinement. While the semantic stream in Dual-SVF effectively decodes essential nucleotide-level features, integrating such models pretrained on massive genomic repositories, such as *Nucleotide Transformer*, *MutBERT,* or *Genos*, can offer a transformative advantage. These models possess the unique capability to capture profound biological representations and long-range genomic dependencies that often elude standard neural architectures. Although the current version of Dual-SVF focuses on establishing a robust baseline for multi-modal feature fusion, integrating such LLM-driven insights remains a priority for future iterations.

In conclusion, Dual-SVF provides a robust, generalizable, and biologically aware solution for SV refinement. Its ability to compensate for limited physical evidence through learned sequence-alignment correlations marks a significant step toward democratization of high-precision LRS. As the field moves toward T2T-level pangenomics and multi-omics integration, Dual-SVF establishes a versatile architecture that can adapt to evolving sequencing technologies and deeper biological insights.

**Key points**

- Dual-SVF addresses high false-positive rates in long-read sequencing structural variant calling by jointly modeling sequence content (semantics) and alignment topology (syntactics) through decoupled encoders.
- A semantic-syntactic mutual correction mechanism integrates genomic priors (e.g. sequence entropy, GC content) via a confidence-gated cross-attention module to adaptively suppress noise and reconcile modality discrepancies.
- A multi-level adaptive constraint loss function enforces cross-modal consistency, prevents modality dominance, and calibrates dynamic prediction boundaries across challenging genomic regions.
- Comprehensive benchmarks across diverse species (human, plant, and yeast) and sequencing platforms (PacBio CLR/HiFiand Oxford Nanopore) demonstrate superior precision, robust cross-species generalizability, and improved Mendelian inheritance fidelity.

## Supplementary Material

Web_Material_bbag448

## Data Availability

The sequencing data and ground truth call sets in this study are listed in supplementary materials. The Dual-SVF code is available at https://github.com/sokolo05/Dual-SVF.
