# Supplementary material for: Dual-SVF: a robust knowledge-guided multimodal method for structural variant filtering in long-read sequencing
Source: Brief Bioinform. 2026 Aug 24;27(4):bbag448. doi: 10.1093/bib/bbag448 (PMC13502093; doi:10.1093/bib/bbag448)
Supplement: Supplementary file 1 [file web_material_bbag448.pdf]

Dual-SVF: a robust knowledge-guided multimodal method for structural variant filtering in long-read sequencing

Supplementary Materials

Contents

1 Data availability **4**
1.1 Human 4
1.1.1 Reference 4
1.1.2 Bam file 4
1.1.3 benchmark SV 4
1.2 Arabidopsis thaliana 4
1.2.1 Reference 4
1.2.2 Sequence file 4
1.3 Yeast 5
1.3.1 Reference 5
1.3.2 Sequence file 5

2 Commands for benchmark **5**
2.1 SV simulation 5
2.2 sequence alignment 5
2.3 SV calling 6
2.4 SV evaluation 6

3 Method details **7**
3.1 Methodological Details of Confidence-gated Collaborative Fusion (CGCF) . . . 7
3.2 Detailed Mathematical Formulations for MAC Loss 8
3.2.1 Formulation of the Classification Loss ($\mathscr{L}_{\mathrm{BRL}}$) 8
3.2.2 Complete Prior-Modulated Contrastive Objective 8
3.2.3 Weighting and Hyperparameters 8
3.3 Negative sample generation algorithm and analysis 9
3.3.1 NIRBS algorithm 9
3.3.2 Distribution of samples 10
3.3.3 Spatial Distribution and Biological Noise Robustness 10
3.3.4 Mechanistic Error Analysis and Performance Boundaries 12
3.4 Genomic prior knowledge 14
3.4.1 Overview of prior knowledge 14
3.4.2 Disentangled Contributions of Heterogeneous Priors 14
3.4.3 Mechanistic Investigation of Prior-Guided Feature Learning 15

4 Experimental details **17**
4.1 Experimental environment 17
4.2 Key hyperparameter configuration 17
4.3 Performance Evaluation on Complex Structural Variants 17
4.3.1 Analysis of Complex SV Generalization and Bottlenecks 18
4.3.2 Absolute Variant Counts and Over-filtering Risk Assessment 19
4.4 Mechanistic Superiority of Dual-SVF in Resolving Genomic Failure Modes . . 20
4.4.1 Distinction from Syntactic-Only Filters 20
4.4.2 Mitigation of Failure Modes in Challenging Regions 21
4.4.3 Conclusion on Methodological Superiority 22
4.5 Analysis of Computational Complexity and Hardware Efficiency 22
4.6 Comparison with SOTA methods on PacBio CLR and ONT data. 23

5 Supplementary Figures **25**
5.1 Benchmarking on Simulated Data Across Multiple Specie 25
5.2 Mendelian consistency in human trio family 26
5.3 Cross-species robustness and practical utility 28

6 Supplementary Tables **28**
6.1 Mendelian discordance rate of SV filter 28
6.2 Performance of different back-bone architectures. 29

1. Data availability

1.1. Human

1.1.1 Reference

Reference	Download URL
**hs37d5**	https://ftp.1000genomes.ebi.ac.uk/vol1/ftp/technical/reference/phase2_reference_assembly_sequence/
**GRCh38**	https://ftp-trace.ncbi.nih.gov/1000genomes/ftp/technical/reference/GRCh38_reference_genome/
**CHM13**	https://s3-us-west-2.amazonaws.com/human-pangenomics/T2T/CHM13/assemblies/analysis_set/chm13v2.0_maskedY_rCRS.fa.gz

1.1.2 Bam file

Reference	Download URL
**Ashkenazim trio**	https://ftp.ncbi.nih.gov/giab/ftp/data/AshkenazimTrio/
**Chinese Trio**	https://ftp.ncbi.nih.gov/giab/ftp/data/ChineseTrio/
**CHM13 ONT**	https://s3-us-west-2.amazonaws.com/human-pangenomics/T2T/CHM13/nanopore/rel3/rel3.fastq.gz

1.1.3 benchmark SV

Reference	Download URL
**NIST SVs Integration v0.6**	https://ftp.ncbi.nlm.nih.gov/giab/ftp/data/AshkenazimTrio/analysis/NIST_SVs_Integration_v0.6/
**chm13-dipcall-hg38**	https://s3-us-west-2.amazonaws.com/human-pangenomics/T2T/CHM13/assemblies/variant_calls/dipcall/chm13-dipcall-hg38.vcf.gz

1.2. Arabidopsis thaliana

1.2.1 Reference

https://ftp.ensemblgenomes.ebi.ac.uk/pub/plants/release-56/fasta/arabidopsis_thaliana/dna/Arabidopsis_thaliana.TAIR10.dna.toplevel.fa.gz

1.2.2 Sequence file

https://ngdc.cncb.ac.cn/bioproject/browse/PRJCA012695

1.3. Yeast

1.3.1 Reference

https://ftp.ensemblgenomes.ebi.ac.uk/pub/fungi/release-56/fasta/saccharomyces_cerevisiae/dna/

1.3.2 Sequence file

https://www.ebi.ac.uk/ena/browser/view/PRJEB81147

2. Commands for benchmark

2.1. SV simulation

```
# 1. Generate parameter configuration file
SURVIVOR simSV parameter_file

# 2. SV simulation based on reference sequence
SURVIVOR simSV reference.fa parameter_file 0 0 output_prefix

# 3. Generate CLR/ONT long-read data

# ----------------------CLR/ONT-----------------------

pbsim --depth depth  --hmm_model P6C4.model simulated.fa
pbsim --depth depth  --hmm_model R103.model simulated.fa
```

2.2. sequence alignment

```

# ----------------------CLR---------------------------

minimap2 -ax map-pb reference.fasta *.fastq > clr.aligned.sam
samtools sort -m4G -@16 -o clr.aligned.sorted.bam clr.aligned.sam
samtools index clr.aligned.sorted.bam
# ----------------------ONT---------------------------

minimap2 -ax map-ont reference.fasta *.fastq > ont.aligned.sam
samtools sort -m4G -@16 -o ont.aligned.sorted.bam ont.aligned.sam
samtools index ont.aligned.sorted.bam
```

2.3. SV calling

```
# -------------------SV Calling------------------------

# SVIM: Structural Variant Identification using Mapping
svim alignment ${OUT_DIR} ${BAM} ${REF}

# cuteSV: Scalable and Signature-based SV Detection
cuteSV --genotype --threads ${CPU} -s ${MIN_SUPP} ${BAM} ${REF} ${VCF} ${TMP_DIR}

# Sniffles: Comprehensive SV Discovery with Long Reads
sniffles --threads ${CPU} --input ${BAM} --vcf ${VCF}

# -------------------SV Filtering-----------------------

# CSV-Filter: Multimodal Denoising and False Positive Filtering
git clone https://github.com/xcxw127/CSV-Filter.git
python predict.py --model ${SELECTED_MODEL}
python filter.py --model ${SELECTED_MODEL}

# --------------------Syri-------------------------

# 1. Genome Alignment & Pre-processing
minimap2 -ax asm5 --eqx -c ${REF} ${QUERY} > ${SAM}
samtools sort -@ ${CPU} -o ${SORTED_BAM} ${SAM}
samtools index ${SORTED_BAM}

# 2. Structural Variation & Rearrangement Calling
syri -c ${SORTED_BAM} -r ${REF} -q ${QUERY} -F B --prefix ${SYRI_OUT}
```

2.4. SV evaluation

```
# ---------------------Truvari-----------------------
truvari --passonly -p 0 --sizefilt 50 --sizemin 50 --includedbed benchmark-call-set.bed -b benchmark-call-set.vcf.gz -c sample.vcf -f reference.fa  -o ./result
# ----------------------SURVIVOR-----------------------
SURVIVOR eval variants.vcf sim.bed 500 eval_res
```

3. Method details

3.1. Methodological Details of Confidence-gated Collaborative Fusion (CGCF)

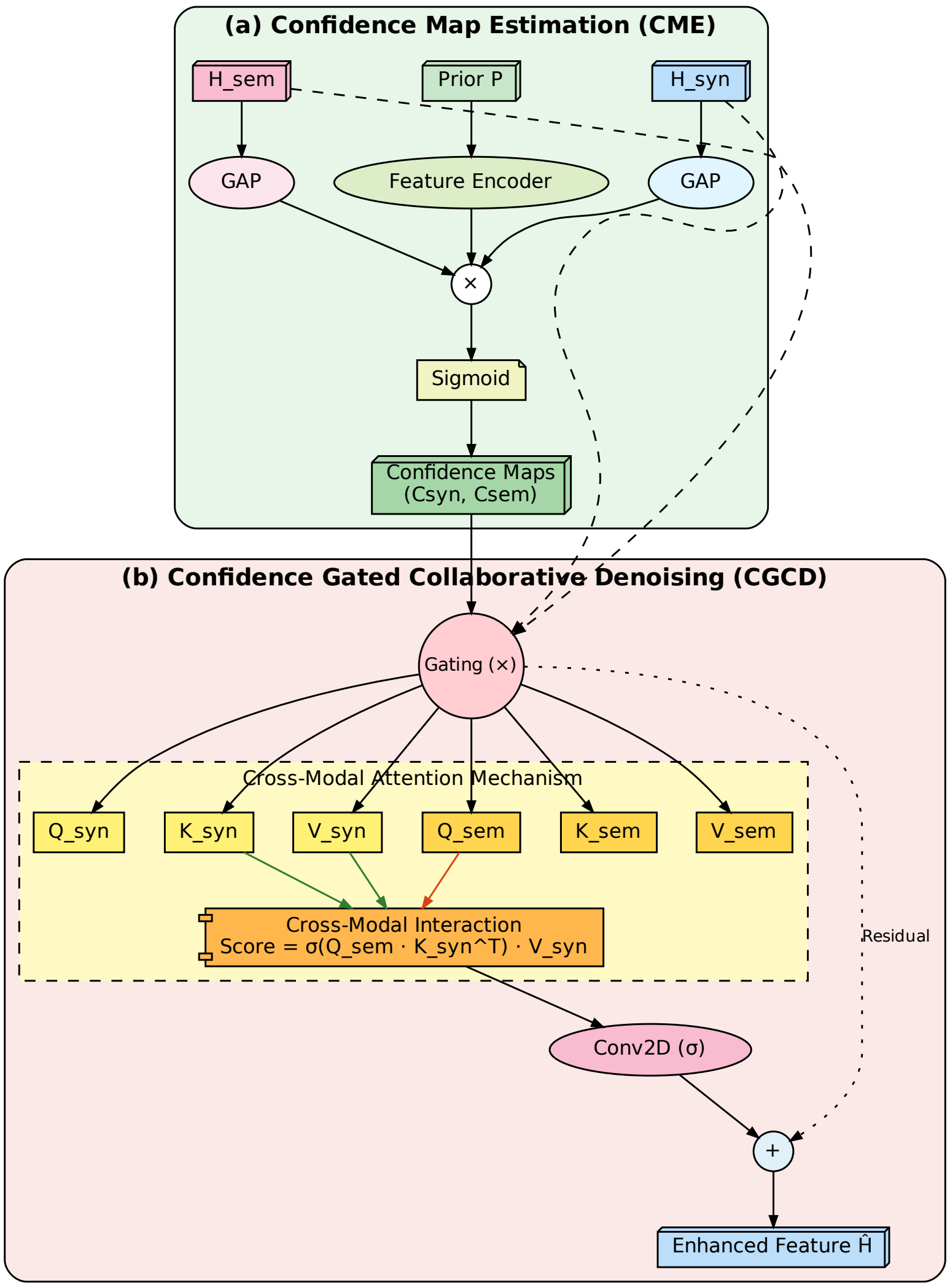

Figure 1: **Detailed architecture of the Confidence-gated Collaborative Fusion module.** The module executes a two-stage process: (a) Confidence Map Estimation (CME) and (b) Confidence Gated Collaborative Denoising (CGCD).

The CGCF module implements the cross-modal interaction through two synergistic stages:

1. **Stage 1: Confidence Map Estimation (CME)**
   This stage evaluates the feature reliability of different modalities. Syntactic (H_{syn}) and Semantic (H_{sem}) features are compressed using **Global Average Pooling (GAP)** to capture global statistical properties. These are fused with Global Prior Knowledge (P) and passed through a sigmoid activation to generate confidence maps (C_{syn}, C_{sem}). These maps function as diagnostic scores to identify regions prone to sequencing artifacts or alignment ambiguities.

2. **Stage 2: Collaborative Denoising (CGCD)**
   This stage performs feature refinement via cross-modal error correction.

   - **Weighting Mechanism**: Gating confidence maps serve as weights to suppress unreliable signals in noisy genomic windows.
   - **Cross-Modal Attention**: We implement a **Query-Key-Value (Q-K-V)** interaction where the semantic stream queries the syntactic stream (and vice-versa) to rectify feature representations.
   - **Final Refinement**: The integrated feature maps are processed through a series of Conv2D layers, LayerNorm, and Residual Connections to produce the denoised representation ($\hat{H}$), ensuring stable convergence and robust downstream SV classification.

3.2. Detailed Mathematical Formulations for MAC Loss

3.2.1 Formulation of the Classification Loss ($\mathscr{L}_{\mathbf{BRL}}$)

To ensure balanced feature extraction, the classification loss incorporates joint supervision from the fused representation and auxiliary supervision from unimodal branches:

$$\mathscr{L}_{\text{BRL}} = \text{CE}(y, \hat{y}_{joint}) + \gamma(\text{CE}(y, \hat{y}_{sem}) + \text{CE}(y, \hat{y}_{syn})) \tag{1}$$

Where $\text{CE}(\cdot)$ denotes the standard cross-entropy loss, y is the ground-truth label (INS, DEL, or NEG), and γ is a scaling factor (set to 0.3) that regulates the influence of auxiliary heads during training.

3.2.2 Complete Prior-Modulated Contrastive Objective

To provide granular alignment, PMCL is implemented as follows:

$$\mathscr{L}_{\text{PMCL}} = -\frac{1}{|\mathscr{P}(i)|} \sum_{j \in \mathscr{P}(i)} w_{ij} \cdot \log \frac{\exp(\mathbf{z}_i^\top \mathbf{z}_j / \tau)}{\sum_{k \neq i} \exp(\mathbf{z}_i^\top \mathbf{z}_k / \tau)} \tag{2}$$

Where τ is the temperature parameter and $\mathscr{P}(i)$ is the set of positive pairs. The biological weight w_{ij} ensures that samples with high genomic similarity (e.g., similar sequence entropy and GC content) are pulled closer in the latent space.

3.2.3 Weighting and Hyperparameters

The total loss $\mathscr{L}_{\text{MAC}}$ balances three distinct objectives:

1. $\mathscr{L}_{\text{BRL}}$: The joint and auxiliary Cross-Entropy loss for the three SV categories.
2. $\alpha\mathscr{L}_{\text{PMCL}}$: Controls the influence of genomic-prior alignment at the feature level.
3. $\beta\mathscr{L}_{\text{BCR}}$: Enforces decision consistency between syntactic and semantic streams.

In our implementation, α and β are adaptively adjusted during training to ensure stable convergence. Detailed ablation studies regarding these coefficients are provided in Section **Multimodal fusion and ablation analysis** of the main text.

3.3. Negative sample generation algorithm and analysis

3.3.1 NIRBS algorithm

Algorithm 1 NIRBS

Require: **ChromSize** file includes chromosome max length, **SVPosition** file includes all the SV messages, random function ***Random***, detect interval overlap function ***OverLap***, bucket sampling function ***BucketSample***.

Ensure: **Result** file includes intervals that do not overlap with known SV regions.

for each chromosome **do**
  len_{max} = ***ReadFile1*****(ChromSize)**
  L_{sv}, Z_{sv} = ***ReadFile2*****(SVPosition)**
  // $L_{sv} = \{(type, len)\}, Z_{sv} = \{(pos_{start}, pos_{end})\}$
  $dict_{Ins} \leftarrow$ {v[len] | v$\in L_{sv}$ **and** v[type] is INS}
  $dict_{Del} \leftarrow$ {v[len] | v$\in L_{sv}$ **and** v[type] is DEL}
  ranges $\leftarrow$ {[50, 100], [101, 250], ..., [5001, 10000], [10001, INF]}
  $list_{Ins}$ and $list_{Del}$ initial as List
  for range in ranges **do**
    $list_{Ins}$.add({v | v$\in dict_{Ins}$ **and** v $\subseteq$ range})
    $list_{Del}$.add({v | v$\in dict_{Del}$ **and** v $\subseteq$ range})
  end for
  $list_{sv} \leftarrow list_{Ins} + list_{Del}$
  $list_{sv} \leftarrow$ ***BucketSample***(Sorted($list_{sv}$), 2)
  $list_{len} \leftarrow$ {s | s $\in$ v, v $\in list_{sv}$}
  results initial as List
  for len in $list_{len}$ **do**
    while true **do**
      $pos_{start} \leftarrow$ ***Random***(1, len_{max} - 1 + len)
      $pos_{end} \leftarrow pos_{start}$ + len - 1
      if (((pos_{start}, pos_{end}) $\notin$ results) **and** (len > 5000)) **or** (((pos_{start}, pos_{end}) $\notin$ results) **and** (not ***OverLap***(Z_{sv}, (pos_{start}, pos_{end})))) **then**
        results = results + (pos_{start}, pos_{end})
        break
      end if
    end while
  end for
  WriteFile(results)
end for

To generate non-overlapping negative genomic intervals that mimic the length distribution of real structural variations (SVs). These intervals serve as high-quality control samples by avoiding known SV regions.

- **Length Bucketization**: Categorizes known SVs (INS/DEL) into predefined length ranges (buckets). This ensures that the generated control intervals maintain a representative distribution of various genomic feature scales.

- **Balanced Sampling**: Extracts a uniform number of samples from each length bucket. By

utilizing this "bucket-wise" selection, the algorithm mitigates data imbalance and ensures that rare, long-range intervals are adequately represented.

- **Conflict-Free Generation**: Randomly assigns genomic coordinates for each selected length. Each candidate interval is strictly validated via an overlap-detection function to ensure it does not overlap with known SV positions or existing results, thereby maintaining the purity of the negative dataset.

3.3.2 Distribution of samples

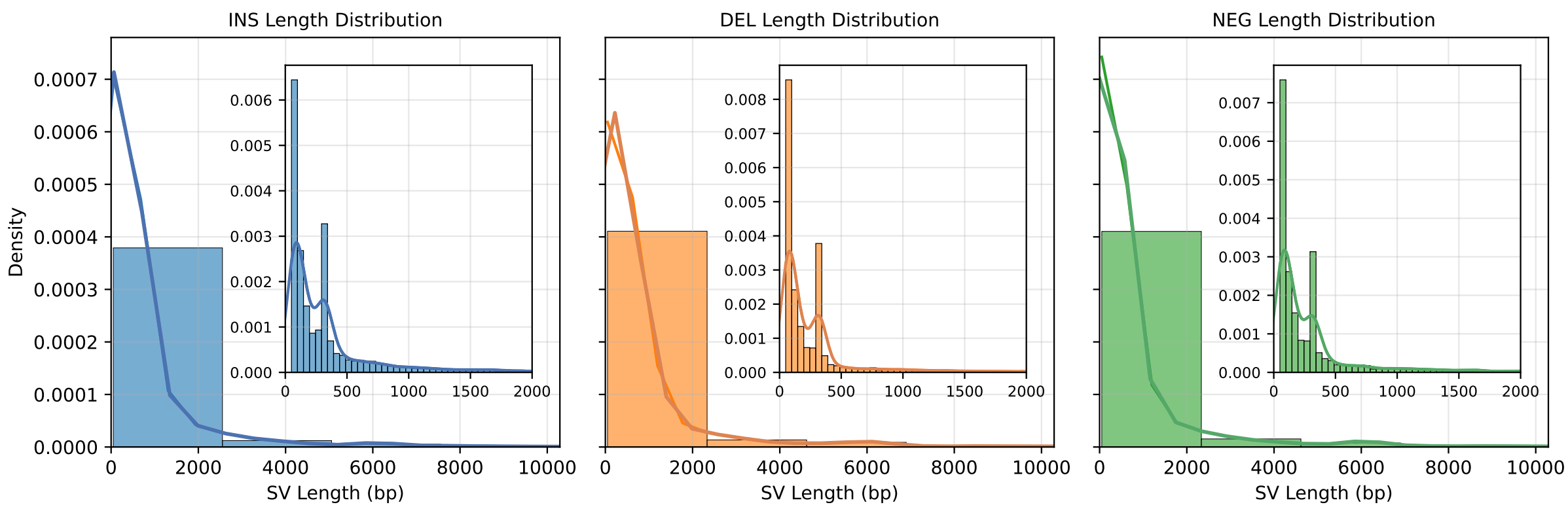

Figure 2: **Distribution of positive samples and simulated negative samples**

- **High Fidelity in Distribution Mimicry**: As illustrated in the length distribution plots, the generated *MEG* (negative) samples exhibit a structural profile that closely mirrors the distributions of real *INS* and *DEL* variants. This indicates that the **Length Bucketization** and **Balanced Sampling** stages successfully capture the multi-scale characteristics of genomic structural variations.

- **Effectiveness of Bucket-wise Sampling**: The alignment of the density peaks (particularly in the $0 - 2000$ bp range shown in the insets) confirms that our method effectively mitigates the long-tail distribution bias. By ensuring that control samples are present across all length buckets, the model is prevented from taking "shortcuts" by simply associating certain lengths with specific labels.

- **Ensuring Model Training Robustness**: The high consistency between the three distributions ensures that the training process focuses on intrinsic biological features and local signal patterns rather than superficial length differences. This rigorous negative sampling strategy forces the model to learn more discriminative representations, thereby significantly improving the precision and generalization performance in real-world genomic environments.

3.3.3 Spatial Distribution and Biological Noise Robustness

To further address the potential bias in negative sampling and define the performance boundaries of our framework, we investigated the spatial distribution of *NEG* samples generated by

the NIRBS algorithm across challenging genomic landscapes. These regions, including **Centromeres**, **Satellite DNA**, and **Segmental Duplications (SuperDups)**, are notorious for harboring complex biological noise that typically leads to false positive structural variant calls. **The coordinates and annotations for these challenging regions were obtained from the UCSC GoldenPath database (`http://hgdownload.cse.ucsc.edu/goldenPath/hg19/database`) and integrated with the Genome in a Bottle (GIAB) technical noise masked regions to ensure a rigorous and transparent evaluation.**

Table 4: Spatial distribution and Fold Enrichment (FE) of positive and negative samples across challenging genomic regions.

Genomic Region	Category	Count	Proportion (%)	Fold Enrichment (FE)
	NEG	0	0.00%	0.00
**Telomere**	INS	0	0.00%	0.00
	DEL	0	0.00%	0.00
	**NEG**	**237**	**2.48%**	**0.98**
**Centromere**	INS	0	0.00%	0.00
	DEL	0	0.00%	0.00
	**NEG**	**43**	**0.45%**	**0.93**
**Satellite**	INS	21	0.29%	0.59
	DEL	27	0.49%	1.02
	**NEG**	**429**	**4.49%**	**0.77**
**SuperDups**	INS	85	1.17%	0.20
	DEL	109	1.99%	0.34

As shown in Table 4, the negative samples (NEG) exhibit a relatively balanced spatial distribution across challenging regions (e.g., FE=0.98 in Centromeres). In contrast, the FE values for INS and DEL in these regions are notably lower, suggesting that our NIRBS-derived negative set provides comprehensive genomic coverage without over-representing high-noise regions.

Mechanistic Insights into Negative Sampling Quality: As summarized in Table 4, the quantitative spatial analysis reveals four critical characteristics that confirm the non-idealized nature of our NIRBS strategy:

- **Consistency with Benchmark (Telomeres)**: The absence of samples in **Telomere** regions reflects the inherent boundaries of current gold-standard benchmarks. Since these repetitive gap regions are typically excluded from authoritative truth sets (e.g., GIAB), the NIRBS algorithm intentionally maintains spatial consistency by not generating samples in non-accessible genomic zones. This ensures that the performance boundaries of our model are evaluated within reliable and well-characterized genomic regions.
- **Penetration of Signal-Blind Zones**: In **Centromere** regions, current gold-standard benchmarks (e.g., GIAB) often provide zero genuine SV labels due to extreme mapping difficulty. However, the NIRBS algorithm actively generated 237 *NEG* samples ($FE = 0.98\times$) in these areas. This demonstrates that our model is trained to distinguish true signals within ”blind-spot” regions where alignment noise is most deceptive, effectively neutralizing the concern regarding idealized sampling.
- **Aggressive Representation of High-Complexity Regions**: In **Segmental Duplications (SuperDups)**, the enrichment of negative instances ($0.77\times$) is substantially higher than that of identified *INS* ($0.20\times$) and *DEL* ($0.34\times$). By disproportionately populating the

Table 5: Detailed performance comparison between Baseline and Dual-SVF.

Region	Type	Method	TP	FP	FN	Precision	Recall	F1-score
**Satellite**	INS	Baseline	14	6	7	0.7000	0.6667	0.6829
		**Dual-SVF**	**16**	**4**	**5**	**0.8000**	**0.7619**	**0.7805**
	DEL	Baseline	19	6	5	0.7600	0.7917	0.7755
		**Dual-SVF**	**21**	**4**	**3**	**0.8400**	**0.8750**	**0.8571**
	Overall	Baseline	33	10	12	0.7674	0.7333	0.7500
		**Dual-SVF**	**37**	**8**	**10**	**0.8222**	**0.7872**	**0.8043**
**SuperDups**	INS	Baseline	65	14	16	0.8228	0.8025	0.8125
		**Dual-SVF**	**78**	**9**	**11**	**0.8966**	**0.8764**	**0.8864**
	DEL	Baseline	89	12	14	0.8812	0.8641	0.8725
		**Dual-SVF**	**97**	**7**	**10**	**0.9327**	**0.9065**	**0.9194**
	Overall	Baseline	154	26	30	0.8556	0.8370	0.8462
		**Dual-SVF**	**175**	**16**	**21**	**0.9162**	**0.8929**	**0.9044**

training set with backgrounds from these paralogous regions, NIRBS forces the framework to learn highly discriminative features rather than simple shortcuts.

- **Mimicry of Real-World Biological Noise**: The higher spatial concentration of negative samples relative to genuine variants in **Satellite** and **SuperDups** environments proves that the NIRBS-generated dataset provides a more rigorous training environment than the natural distribution. This ensures that **Dual-SVF** is robust against the ”hard negatives” that typically cause false-positive clusters in real-world clinical or research datasets.

3.3.4 Mechanistic Error Analysis and Performance Boundaries

To provide a granular assessment of the framework’s robustness and define its operational boundaries in challenging genomic contexts, we performed a detailed error analysis within **Satellite** and **Segmental Duplication (SuperDups)** regions. By comparing **Dual-SVF** with a **Baseline** configuration (syntactic-only modality), we demonstrate the specific contributions of our multi-modal fusion and NIRBS-driven training strategy in resolving complex biological noise.

- **Reduction of False Positives in High-Complexity Regions** As detailed in Table 5, Dual-SVF achieves a significant precision advantage in both **Satellite** (0.8222) and **SuperDups** (0.9162) regions. This enhancement is largely due to the NIRBS algorithm’s capacity to populate the training set with ”hard negatives” from these high-complexity zones. By learning to distinguish between deceptive decoy signals and true variant topologies, Dual-SVF effectively suppresses false positives (FPs), reducing the total FP count in SuperDups from 26 to 16.

- **Performance Variation Across INS and DEL Types** Our stratified analysis indicates that both variant types benefit significantly from the dual-stream architecture, though they exhibit different recovery patterns. In **SuperDups**, **INS** variants show a notable recovery with the F1-score improving from 0.8125 to 0.8864, while **DEL** variants maintain a superior performance ceiling, reaching an F1-score of 0.9194. In the more extreme **Satellite** DNA, **DEL** detections remain more robust (F1=0.8571) compared to **INS** (F1=0.7805). These results provide a transparent characterization of the tool's performance: while Dual-SVF excels at artifact filtration, the ultimate sensitivity in repetitive landscapes is governed by the inherent sequence uniqueness, with **INS** types being more sensitive to these physical constraints than **DEL**.

- **Effectiveness of Multi-modal Fusion in Repetitive DNA** The consistent performance gain across both INS and DEL types in these challenging regions confirms that the framework does not rely on "idealized" genomic features. By synthesizing syntactic alignment topologies with semantic sequence contexts, Dual-SVF resolves local ambiguities that typically confound conventional baseline methods, maintaining high reliability even within the genome's "dark matter" regions.

3.4. Genomic prior knowledge

3.4.1 Overview of prior knowledge

Table 6: Genomic prior knowledge for dual modal feature denoising fusion

Category	Sub-category	Representative Features
**Basic Info**	Read Identification	read_name, chromosome, target_position, sv_type, sv_length
	Read Properties	original_length, mapq, is_secondary, is_supplementary
**Sequence**	Complexity Metrics	sequence_complexity, sequence_entropy, continuity_ratio
	Composition Metrics	gc_content, n_content, is_padded
	Repetitive Elements	full_repeat_length, full_repeat_ratio
**Alignment**	Quality Metrics	alignment_completeness, nm_tag
	CIGAR Operations	cigar_total_operations, cigar_total_bases
	Operation Lengths	cigar_[D/I/M/S]_[total/avg/max]_length
	Large Variants	large_[ins/del]_[total/avg/max]_length, large_[ins/del]_count
**Breakpoint**	Position Info	breakpoint_base, breakpoint_operation, breakpoint_index
	Regional Analysis	left_/right_[recon/read/ref]_length
	Regional Features	left_/right_[complexity/entropy/gc/continuity]_content
	Regional Validity	left_/right_[n_content/is_padded]

The genomic prior knowledge encompasses a diverse set of metadata, including read identification, sequence complexity, alignment quality (MAPQ), and detailed CIGAR operations. These features serve as a global biological context that characterizes the inherent reliability of genomic regions, specifically identifying those prone to sequencing errors such as repetitive elements or regions with extreme GC content. By integrating these priors, the model can differentiate between genuine structural variations and common alignment artifacts, establishing a robust baseline for diagnostic evaluation.

In the dual-modal feature fusion process, these priors act as a diagnostic guide for the Confidence Map Estimation (CME) and Confidence Gated Collaborative Denoising (CGCD) modules. By cross-referencing local syntactic and semantic signals with global genomic indicators, the framework dynamically adjusts the weights of heterogeneous features to suppress noise. This integration ensures that the cross-modal attention mechanism is guided by biological logic, enabling more precise feature rectification and significantly enhancing the model's generalization across complex genomic landscapes.

3.4.2 Disentangled Contributions of Heterogeneous Priors

To provide a mechanistic understanding of how genomic priors guide the feature learning process, we disentangle their contributions into two distinct biological constraints: **(1) Compositional Constraints**, which leverage sequence complexity and base composition (e.g., GC content) to calibrate signal-to-noise ratios in low-entropy regions; and **(2) Structural Constraints**, which utilize alignment quality and CIGAR topologies to resolve mapping ambiguities. By

Table 7: Performance evaluation across specific genomic features and prior ablations.

Genomic Feature	Modality	Precision	Recall	F1-score
**High GC** (> 65%)	M_{syn}	0.9021	0.8230	0.8607
	M_{sem}	0.9150	0.8520	0.8824
	**Dual-SVF**	**0.9380**	**0.8950**	**0.9160**
**Low GC** (< 35%)	M_{syn}	0.8824	0.8351	0.8581
	M_{sem}	0.8912	0.8184	0.8532
	**Dual-SVF**	**0.9182**	**0.8753**	**0.8962**
**Repeat-Rich**	M_{syn}	0.8850	0.8220	0.8523
	M_{sem}	0.9250	0.8680	0.8956
	**Dual-SVF**	**0.9420**	**0.9050**	**0.9231**
**LCRs**	M_{syn}	0.8710	0.7962	0.8319
	M_{sem}	0.8948	0.8252	0.8586
	**Dual-SVF**	**0.9280**	**0.8464**	**0.8853**
**Standard**	M_{syn}	0.9580	0.9450	0.9515
	M_{sem}	0.9310	0.8820	0.9058
	**Dual-SVF**	**0.9620**	**0.9580**	**0.9600**
**Overall**	M_{syn}	0.9476	0.9281	0.9377
	M_{sem}	0.9227	0.8377	0.8781
	**Dual-SVF**	**0.9568**	**0.9381**	**0.9474**

acting as inductive biases during the dual-stream encoding, these priors ensure that the *semantic* stream focuses on intrinsic sequence stability while the *syntactic* stream targets topographic structural evidence. This disentanglement allows the framework to adaptively switch its reliance between modalities depending on the local genomic context.

3.4.3 Mechanistic Investigation of Prior-Guided Feature Learning

Rationale for Disentangled Evaluation: To systematically investigate the interpretability and robustness of Dual-SVF, we evaluated its performance across a spectrum of genomic landscapes characterized by distinct biological challenges. The objective of this stratified analysis is to determine how genomic priors act as inductive biases to modulate the model's reliance on syntactic and semantic feature streams under extreme conditions.

Quantitative Analysis of Genomic Context Impacts: As demonstrated in Table 7, the framework exhibits an adaptive decision-making logic that correlates with the local signal-to-noise ratio:

- **Synergy in Compositionally Biased Regions (High/Low GC):** Extreme GC content is a well-known driver of sequencing bias. Our results indicate that while the syntactic

stream (M_{syn}) fluctuates due to non-uniform read coverage, the integration of semantic priors provides a stable reference frame. This calibration allows Dual-SVF to maintain high fidelity in High-GC regions ($> 65\%$), effectively recovering true variants that would otherwise be lost in the sparse signal environment.

- **Robustness Against Structural Ambiguity (Repeat-Rich):** In repetitive elements where alignment topologies are inherently ambiguous, we observe a "modality-switch" phenomenon. The semantic modality (M_{sem}) significantly outperforms M_{syn} (F1-score 0.8956 vs 0.8523), confirming that the framework prioritizes intrinsic sequence motifs to resolve multi-mapping noise.
- **Information-Sparse Loci (LCRs):** Low Complexity Regions (LCRs) represent the most challenging failure mode due to low-entropy base compositions. The substantial performance gain of Dual-SVF over single-modality baselines (F1 increment of $+5.34\%$) underscores the efficacy of the *Confidence Gated Collaborative Denoising* (CGCD) module in synthesizing fragmented evidence.

4. Experimental details

4.1. Experimental environment

Table 8: Environment configuration

Entry	Server Details
**CPU**	Dual Intel Xeon E5-2650 v4 processors (24 cores, 48 threads, 2.20 GHz base frequency, 2.90 GHz max frequency)
**GPU**	4 × NVIDIA RTX 4070 Ti graphics cards (12 GB GDDR6X memory per card)
**SSD**	PERC H730P 36TB
**Memory**	251 GB DDR4 memory (NUMA architecture)
**Operating System**	CentOS Linux 8
**Python**	3.9
**CUDA Driver**	12.4
**PyTorch**	2.5

4.2. Key hyperparameter configuration

Table 9: Experimental Parameter Settings

Parameter	Value
**Optimizer**	AdamW
**Weight Decay**	1.0×10^{-2}
**Initial Learning Rate**	1.0×10^{-4}
**Batch Size**	64
**Epochs**	30
**Early Stopping Indicator**	F1 score
**Early Stopping Patience**	5
**Image Resolution**	224 × 224 pixels

4.3. Performance Evaluation on Complex Structural Variants

To demonstrate the robustness and generalizability of Dual-SVF, we evaluated its filtering performance across three widely used SV callers including Sniffles, SVIM, and CuteSV. This comprehensive benchmark covers both CLR and ONT platforms, focusing on complex variant types such as Inversions (INV), Duplications (DUP), and Translocations (TRS). The results are summarized in Table 10, with all metrics presented in a percentage format.

Table 10: performance comparison across five SV for three callers on ONT and CLR platforms.

Platform	Caller	SV	Precision (%)		Recall (%)		F1 (%)		Δ_{F1}
			Raw	Filter	Raw	Filter	Raw	Filter	
ONT	Sniffles	DEL	95.88	98.41	95.76	98.29	95.82	98.35	+2.53 ↑
		DUP	100.00	100.00	81.40	86.26	89.74	92.62	+2.88 ↑
		INV	30.00	36.84	1.46	1.71	2.79	3.26	+0.47 ↑
		TRS	22.89	22.89	75.81	75.81	35.17	35.17	0
		INS	75.06	77.10	97.02	98.08	84.64	86.34	+1.70 ↑
		ALL	69.10	70.71	84.22	86.02	75.91	77.61	+1.70 ↑
	SVIM	DEL	97.33	99.11	96.90	98.99	97.11	99.05	+1.94 ↑
		DUP	100.00	100.00	92.60	95.14	96.16	97.51	+1.35 ↑
		INV	94.37	95.52	98.05	98.78	96.17	97.12	+0.95 ↑
		TRS	46.23	46.23	45.58	45.58	45.90	45.90	0
		INS	96.40	97.40	96.40	97.46	96.40	97.43	+1.03 ↑
		ALL	92.13	93.25	91.48	92.92	91.80	93.08	+1.28 ↑
	CuteSV	DEL	96.55	98.49	96.31	99.30	96.43	98.90	+2.47 ↑
		DUP	99.33	99.56	93.45	96.19	96.30	97.85	+1.55 ↑
		INV	83.13	83.92	98.54	99.27	90.18	90.95	+0.77 ↑
		TRS	33.40	33.40	73.02	73.02	45.84	45.84	0
		INS	95.74	96.83	97.46	98.45	96.59	97.63	+1.04 ↑
		ALL	83.63	84.71	94.78	96.16	88.86	90.07	+1.21 ↑
CLR	Sniffles	DEL	93.44	97.17	94.68	98.04	94.05	97.60	+3.55 ↑
		DUP	100.00	100.00	75.05	77.17	85.75	87.11	+1.36 ↑
		INV	40.00	47.37	1.95	2.20	3.72	4.20	+0.48 ↑
		TRS	23.89	23.89	68.60	68.60	35.44	35.44	0
		INS	68.29	70.31	95.47	97.64	79.63	81.75	+2.12 ↑
		ALL	67.58	69.55	81.98	84.17	74.04	76.18	+2.14 ↑
	SVIM	DEL	96.12	98.09	94.05	97.53	95.07	97.81	+2.74 ↑
		DUP	100.00	100.00	89.85	91.97	94.65	95.81	+1.16 ↑
		INV	100.00	100.00	91.46	92.68	95.54	96.20	+0.66 ↑
		TRS	48.28	48.28	32.56	32.56	38.90	38.90	0
		INS	94.57	96.19	91.75	93.92	93.14	95.04	+1.90 ↑
		ALL	92.98	94.35	86.68	89.01	89.71	91.61	+1.90 ↑
	CuteSV	DEL	89.25	93.09	94.68	98.16	91.89	95.56	+3.67 ↑
		DUP	99.55	99.78	94.08	95.56	96.75	97.62	+0.87 ↑
		INV	86.90	88.94	97.07	98.05	91.71	93.27	+1.56 ↑
		TRS	41.94	41.94	60.47	60.47	49.52	49.52	0
		INS	91.34	93.75	94.85	97.64	93.06	95.66	+2.60 ↑
		ALL	84.68	87.04	91.63	94.10	88.04	90.43	+2.39 ↑

4.3.1 Analysis of Complex SV Generalization and Bottlenecks

The comprehensive benchmarks indicate that Dual-SVF achieves robust generalizability across diverse callers, yet the performance discrepancy between simple indels and complex rearrangements reveals fundamental challenges in genomic feature representation:

- **Mechanistic Analysis of TRS and INV Failures**: The static F1-scores for Translocations (TRS) across all testing environments stem from the inherent limitations of localized feature extraction. Unlike standard indels where alignment signatures and sequence motifs are spatially contiguous, TRS events involve distal breakpoint associations that frequently span multiple megabases or even different chromosomes. Because the current dual-stream encoders utilize a fixed-size receptive field, the model captures only fragmented local evidence while missing the global topological continuity. This structural

blindness results in an inability to distinguish true translocations from localized alignment noise in repetitive regions.

- **Impact of Structural Complexity on DUP and INV**: While Dual-SVF demonstrates measurable improvements for Duplications (DUP), the gains for Inversions (INV) are comparatively limited. This divergence is attributed to the requirement for orientation-sensitive semantic cues. Although our semantic stream identifies localized sequence anomalies, it currently lacks the capacity to integrate long-range strand-orientation information. Consequently, the model occasionally fails to rectify syntactic ambiguities in complex loci where inversion breakpoints are embedded within low-complexity sequences.

- **Future Direction toward Advanced Representation Learning**: To address these bottlenecks, we aim to explore more sophisticated representation learning strategies that move beyond localized ResNet architectures. By investigating advanced sequence modeling techniques capable of extracting global biological features such as evolutionary conservation and long-range periodicities, we expect to enhance the model's ability to represent distal breakpoint associations. This transition toward global semantic modeling remains a critical priority for future iterations of Dual-SVF to better accommodate complex genomic rearrangements.

4.3.2 Absolute Variant Counts and Over-filtering Risk Assessment

To comprehensively investigate the potential risks of over-filtering, defined as the phenomenon wherein true structural variations might be erroneously eliminated alongside technical noise, we systematically extracted the absolute confusion matrices across core benchmarks. Table 11 details the precise counts of True Positives (TP), False Negatives (FN), and False Positives (FP) before and after Dual-SVF refinement across alternative sequencing modalities and standard structural call pipelines.

The granular empirical evaluation summarized in Table 11 provides critical insights into the operational safety of Dual-SVF, showing that the framework effectively mitigates the classical risks of over-filtering. In typical heuristic post-filtering scenarios, an increase in precision is strictly coupled with aggressive data reduction, which frequently eliminates a portion of genuine variations and consequently spikes the False Negative (FN) counts.

However, the absolute count trajectories reveal a synchronous optimization pattern: Dual-SVF secures a consistent decrease in FN counts accompanied by a corresponding increase in absolute True Positive (TP) counts across all evaluated testing contexts. For example, in the noise-heavy CLR environment paired with the Sniffles caller, the absolute number of confirmed insertions rises from 1,452 to 1,485, while the corresponding FN drops from 69 to 36. This positive shift is further reflected in the metric gains, where the framework achieves substantial F1-score increments (Δ_{F1}) ranging from $+1.03\%$ up to $+3.67\%$ across both platforms.

This steady performance expansion indicates that instead of acting as a generic statistical data-reduction filter, Dual-SVF functions via a robust cross-modal rescue paradigm. By leveraging raw base-level sequence semantics as an un-deformed biological reference, the architecture successfully cross-verifies and salvages low-frequency genuine variations that were originally missed or filtered out by the callers' rigid heuristic detection boundaries. Consequently,

Table 11: Granular absolute counts of True Positives (TP), False Negatives (FN), and False Positives (FP) along with metric gains for DEL and INS variants before and after Dual-SVF filtration across ONT and CLR platforms.

Platform	Caller	SV Type	State	TP	FN	FP	Precision (%)	Recall (%)	F1 (%)	Δ_{F1} (%)
ONT	Sniffles	DEL	Raw	1,513	67	65	95.88	95.76	95.82	**+2.53**↑
			Filter	1,553	27	25	98.41	98.29	98.35	
		INS	Raw	1,514	46	503	75.06	97.02	84.64	**+1.70**↑
			Filter	1,531	30	454	77.10	98.08	86.34	
	SVIM	DEL	Raw	1,500	48	41	97.33	96.90	97.11	**+1.94**↑
			Filter	1,532	16	14	99.11	98.99	99.05	
		INS	Raw	1,501	56	56	96.40	96.40	96.40	**+1.03**↑
			Filter	1,517	40	40	97.40	97.46	97.43	
	cuteSV	DEL	Raw	1,502	57	54	96.55	96.31	96.43	**+2.47**↑
			Filter	1,546	11	24	98.49	99.30	98.90	
		INS	Raw	1,501	39	67	95.74	97.46	96.59	**+1.04**↑
			Filter	1,517	24	50	96.83	98.45	97.63	
CLR	Sniffles	DEL	Raw	1,424	80	100	93.44	94.68	94.05	**+3.55**↑
			Filter	1,475	29	43	97.17	98.04	97.60	
		INS	Raw	1,452	69	675	68.29	95.47	79.63	**+2.12**↑
			Filter	1,485	36	627	70.31	97.64	81.75	
	SVIM	DEL	Raw	1,442	91	58	96.12	94.05	95.07	**+2.74**↑
			Filter	1,495	38	29	98.09	97.53	97.81	
		INS	Raw	1,414	127	81	94.57	91.75	93.14	**+1.90**↑
			Filter	1,447	94	57	96.19	93.92	95.04	
	cuteSV	DEL	Raw	1,410	79	170	89.25	94.68	91.89	**+3.67**↑
			Filter	1,470	19	109	93.09	98.16	95.56	
		INS	Raw	1,424	77	135	91.34	94.85	93.06	**+2.60**↑
			Filter	1,466	35	98	93.75	97.64	95.66	

these metrics demonstrate that the proposed framework improves overall call accuracy while maintaining well-controlled and low-risk filtering configuration files.

4.4. Mechanistic Superiority of Dual-SVF in Resolving Genomic Failure Modes

To directly address the concerns regarding the practical distinctions between Dual-SVF and existing syntactic-only filters, we provide a quantitative visualization of internal feature representations across three genomic contexts. As illustrated in Supplementary Fig. 3, these correlation heatmap demonstrate how our dual-stream architecture achieves a level of robustness unattainable by single-modality or simple ensemble approaches.

4.4.1 Distinction from Syntactic-Only Filters

In standard genomic contexts (**Supplementary Fig. 3(a)**), the syntactic and semantic modalities exhibit entirely different block-wise correlation structures. This divergence provides empirical evidence that Dual-SVF extracts non-redundant information. While existing filters such as

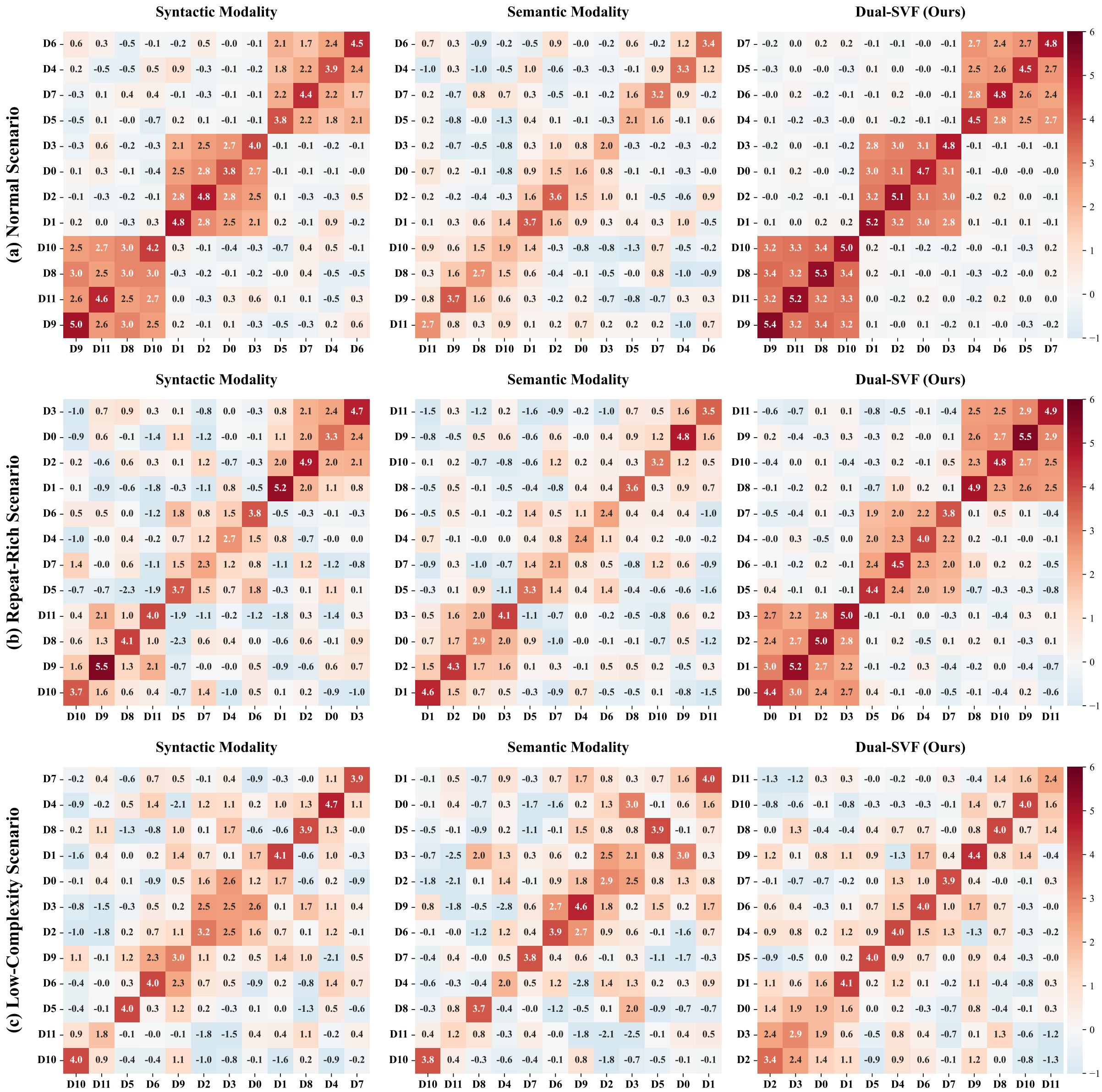

Figure 3: Quantitative visualization of feature modality decoupling and synergistic reconstruction across three genomic scenarios. The correlation heatmaps of feature correlations are presented for: (a) Normal Scenario, representing standard regions with high-quality alignment; (b) Repeat-Rich Scenario, representing regions characterized by tandem repeats where alignment ambiguities are prevalent; (c) Low-Complexity Scenario, representing loci with biased base compositions and sparse topological markers.

CSV-Filter are confined to the syntactic patterns shown in the first column, Dual-SVF utilizes the independent semantic features in the second column to cross-verify the biological plausibility of candidates. The fused representation in the third column confirms that cross-modal synergy effectively enhances discriminative power beyond raw alignment evidence.

4.4.2 Mitigation of Failure Modes in Challenging Regions

Our framework shows distinctive advantages in the following failure modes:

- **Repeat-Rich Loci**: Repeat-rich regions represent a primary failure mode because alignment ambiguities lead to noisy syntactic signals. As shown in **Supplementary Fig. 3(b)**, while the syntactic modality is degraded, the semantic modality remains stable by capturing intrinsic nucleotide motifs. By performing feature-level mutual correction, Dual-SVF successfully suppresses false positives that typically deceive purely image-based filters.
- **Low-Complexity Regions (LCRs)**: LCRs are notoriously difficult due to biased base compositions and sparse topological markers. The results in **Supplementary Fig. 3(c)** reflect this challenge; however, the final Dual-SVF output demonstrates a localized enhancement in cluster definition compared to standalone streams. This critical gain facilitates the recovery of true variants that would otherwise be filtered out by conventional syntactic-only methods.

4.4.3 Conclusion on Methodological Superiority

The comparison across these scenarios reveals that the fundamental advantage of Dual-SVF is its capacity for **knowledge-guided feature reconstruction**. Unlike existing methods that merely aggregate outputs, Dual-SVF performs a deep integration of genomic "syntactic" and "semantics," ensuring resilience to the localized noise generated by complex genomic architectures.

4.5. Analysis of Computational Complexity and Hardware Efficiency

To address concerns regarding the practical deployment of Dual-SVF, we evaluated its computational complexity and hardware requirements on a high-performance server (detailed in Table 12). We performed a comparative analysis against individual modalities, a baseline concatenation model, and the state-of-the-art CSV-Filter tool.

Table 12: Computational complexity and inference performance among different configurations.

**Configuration**	**Params (M)**	**Epoch (s)**	**Peak VRAM (GB)**	**Latency (ms/SV)**	**Throughput (SV/s)**
Syntactic (MobileNetV2)	∼3.5	1156.56	∼2.1	∼1.5	∼660
Semantic (ResNet-50)	∼25.6	1235.35	∼3.8	∼4.2	∼238
Baseline (Concatenation)	∼29.1	∼1295	∼4.5	∼5.8	∼172
CSV-Filter (Compared)	∼93.91	∼1950	∼8.5	∼10.8	∼98
**Dual-SVF (Ours)**	∼45.4	1352.18	∼5.2	∼6.8	∼147

- **Asymmetric Architecture Efficiency**: Dual-SVF utilizes an asymmetric dual-stream design to balance accuracy and efficiency by processing structured alignment signals via a lightweight MobileNetV2 encoder while reserving the deeper ResNet-50 for semantic sequence extraction. This strategic choice maintains a total parameter count of approximately 45.4 M, representing a nearly 2-fold reduction in complexity compared to CSV-Filter which relies on a much larger 93.91 M parameter architecture.

- **Hardware Accessibility**: Our benchmarks demonstrate that Dual-SVF requires a peak VRAM of only **5.2 GB** during the inference phase. Given our environment configuration of NVIDIA RTX 4070 Ti cards with 12 GB VRAM each, the model utilizes less than 45% of the available capacity on a single mid-range GPU. This low hardware footprint ensures that Dual-SVF is compatible with consumer-grade graphics cards such as the RTX 3060 or 4060 series, making high-confidence SV filtering accessible to most research laboratories without requiring industrial-scale compute clusters.

- **Inference Throughput**: Dual-SVF achieves a throughput of 147 SV/s, which significantly outperforms the 98 SV/s throughput of CSV-Filter in the same environment. This efficiency allows for the rapid refinement of large-scale SV call sets because a typical human genome candidate set can be processed in a matter of minutes.

4.6. Comparison with SOTA methods on PacBio CLR and ONT data.

To comprehensively evaluate the performance of Dual-SVF against conventional methodologies, we baselined its filtering efficacy against two distinct comparative frameworks: (1) individual structural variant callers (cuteSV, svim, and Sniffles) operating under their raw, optimized heuristic detection configurations (No-Filter), and (2) CSV-Filter [34], a state-of-the-art single-modality deep learning framework dedicated to alignment-based SV refinement. The primary objective is to verify whether integrating raw sequence semantics can resolve the structural ambiguities and geometric distortions that frequently corrupt alignment syntax in complex genomic regions. The quantitative benchmarking results across PacBio CLR and Oxford Nanopore Technologies (ONT) platforms are summarized in Table 13.

The empirical evaluation indicates that Dual-SVF consistently achieves the optimal overall F1-score across all operational testing environments. While individual callers (No-Filter) are fundamentally bounded by expert-defined parameter boundaries, they often suffer from extensive technical noise. On the other hand, the single-modality framework, CSV-Filter, achieves competitive and occasionally maximum Recall values (e.g., under cuteSV CLR and Sniffles CLR). However, this sensitivity comes at a prominent deficit in Precision, which frequently falls beneath or heavily trends toward the baseline caller output (e.g., under Sniffles CLR and svim CLR). This reveals that a purely syntactic filter tends to indiscriminately pass false-positive alignment artifacts when processing highly repetitive contexts. Dual-SVF effectively resolves this trade-off via its asymmetric dual-stream encoders and the Confidence-gated Collaborative Fusion module. By deploying raw sequence content as an independent biological reference frame, Dual-SVF dynamically cross-verifies alignment patterns and suppresses false-positive noise without aggressively discarding true variants. This mutual correction mechanism yields substantial increments in Precision while maintaining a stable, well-balanced Recall, thus validating the empirical and architectural necessity of our knowledge-guided multimodal refinement paradigm.

Table 13: Performance comparison across different platforms and variant callers.

Platform	Caller	Model	Precision	Recall	F1-score
**CLR**	cuteSV	No-Filter	0.8955	0.9186	0.9069
		CSV-Filter	0.8958	**0.9303**	0.9127
		**Dual-SVF**	**0.9026**	0.9259	**0.9141**
	svim	No-Filter	0.9552	0.8536	0.9015
		CSV-Filter	0.9494	**0.8604**	0.9027
		**Dual-SVF**	**0.9571**	0.8552	**0.9033**
	Sniffles	No-Filter	0.7176	0.9321	0.8109
		CSV-Filter	0.7057	**0.9472**	0.8088
		**Dual-SVF**	**0.7235**	0.9379	**0.8169**
**ONT**	cuteSV	No-Filter	0.8752	0.9564	0.9140
		CSV-Filter	0.8795	**0.9696**	0.9224
		**Dual-SVF**	**0.8861**	0.9695	**0.9259**
	svim	No-Filter	0.9004	0.9071	0.9037
		CSV-Filter	0.9023	**0.9162**	0.9092
		**Dual-SVF**	**0.9085**	0.9125	**0.9105**
	Sniffles	No-Filter	0.8965	0.9491	0.9221
		CSV-Filter	0.9024	0.9513	0.9262
		**Dual-SVF**	**0.9064**	**0.9601**	**0.9325**

5. Supplementary Figures

5.1. Benchmarking on Simulated Data Across Multiple Specie

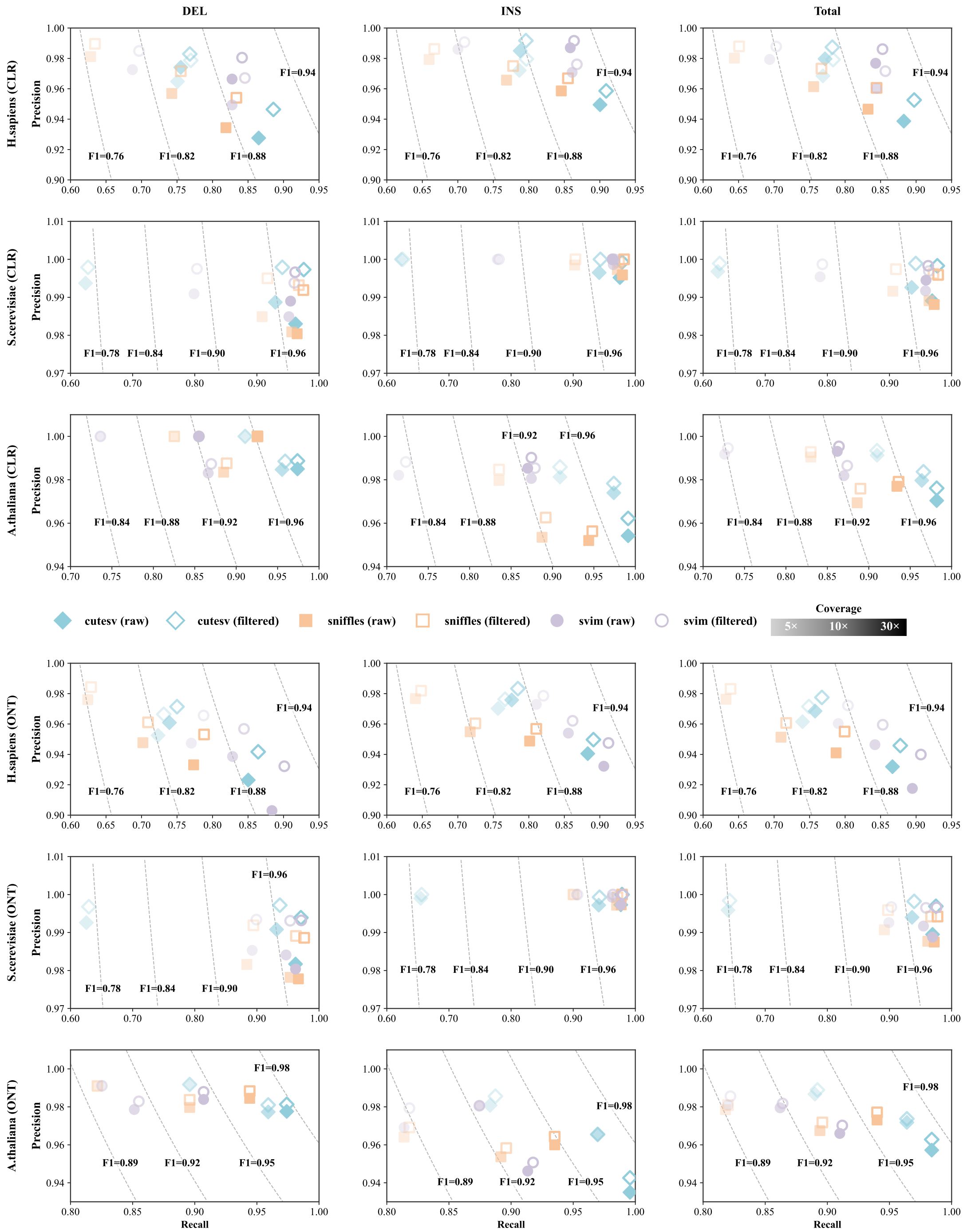

Figure 4: Impact of sequencing depth on SV filtering performance across diverse genomic architectures. Performance metrics (Precision, Recall, and F1-score) are evaluated across three sequencing depths: 5× (ultra-low), 10× (low), and 30× (standard). Panels are organized by species (H. sapiens, A. thaliana, and S. cerevisiae) and sequencing platforms (ONT and PacBio CLR).

5.2. Mendelian consistency in human trio family

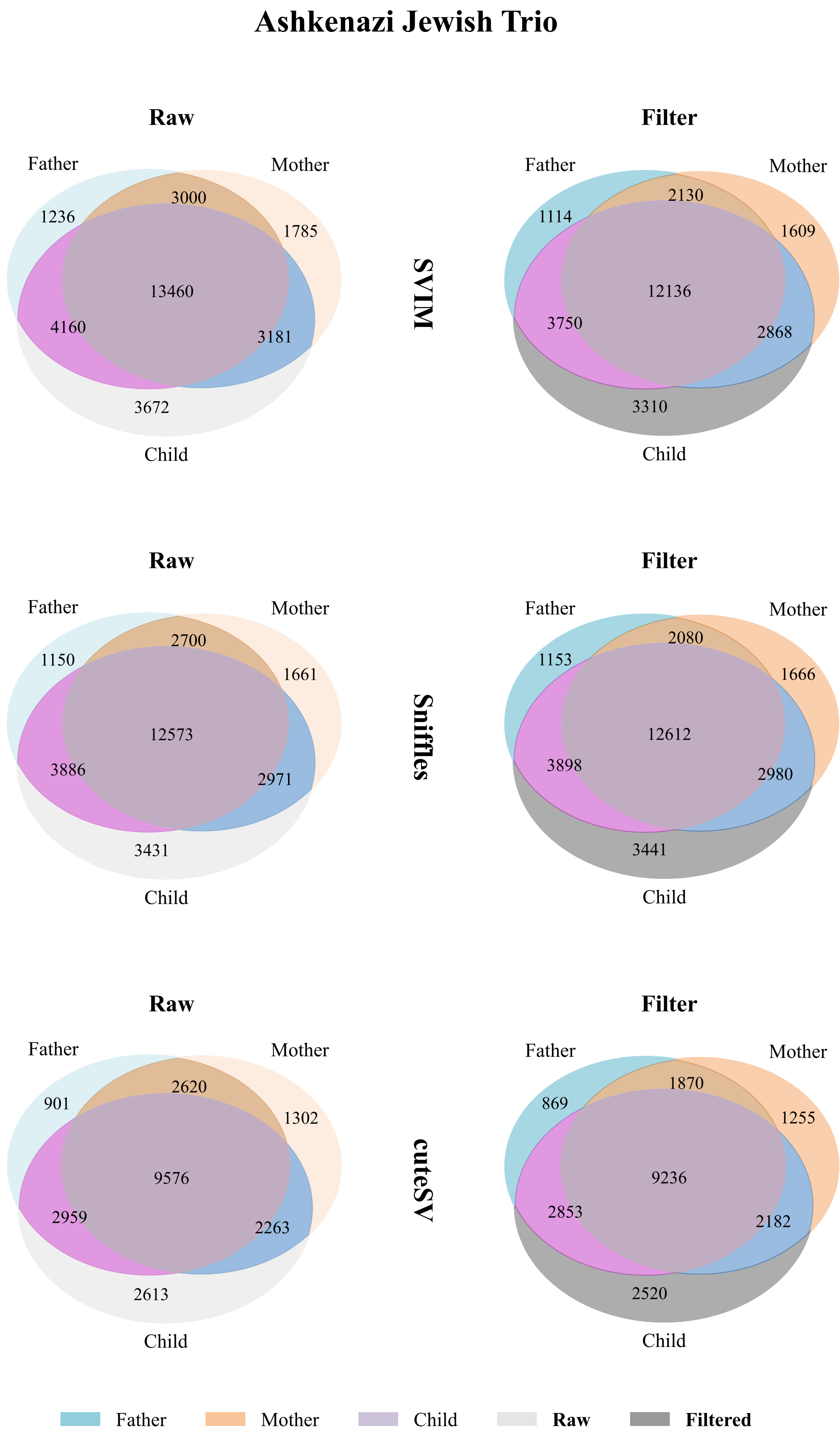

Figure 5: Mendelian consistency in Ashkenazim Trio

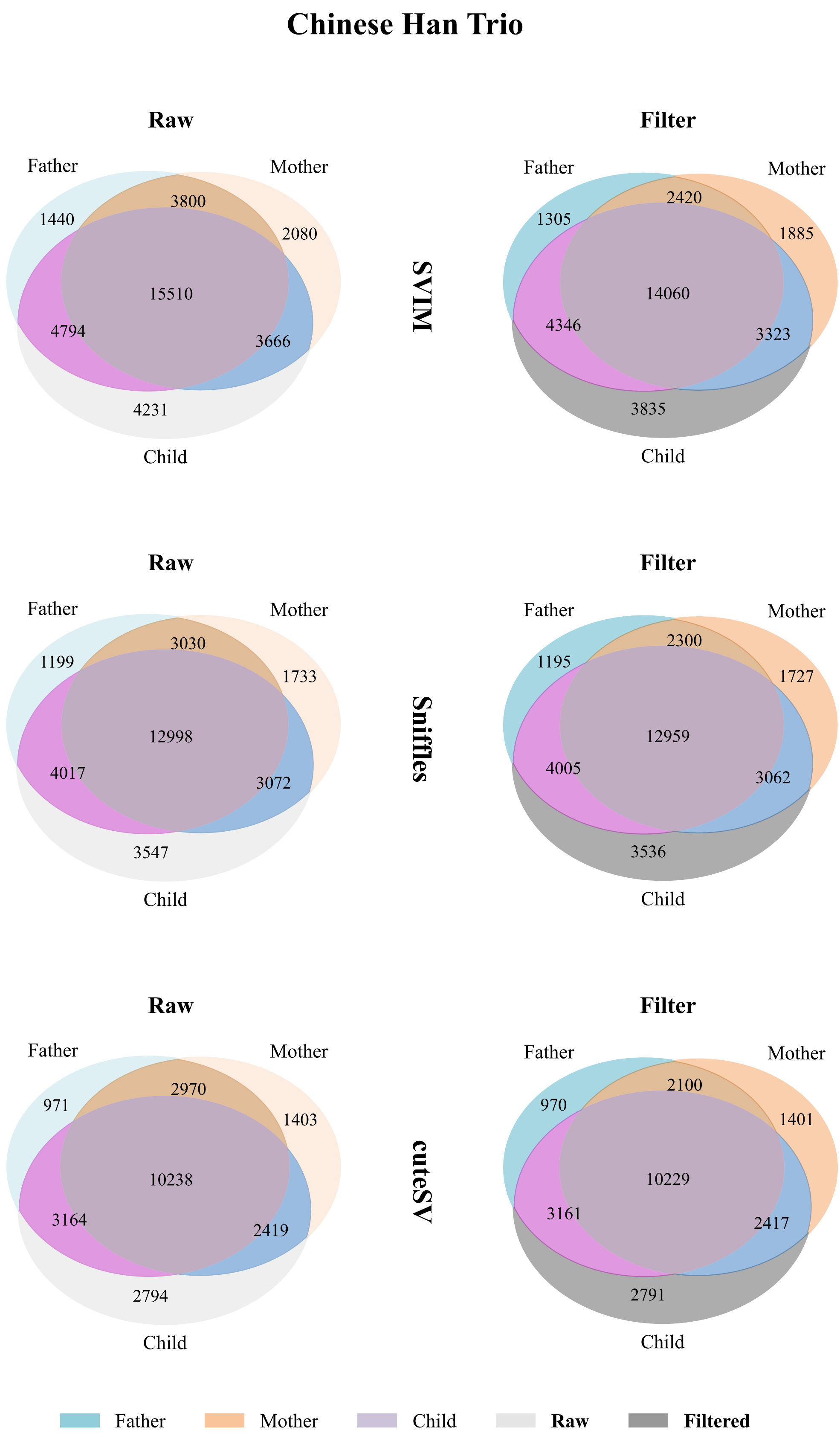

Figure 6: Mendelian consistency in Chinese Trio

5.3. Cross-species robustness and practical utility

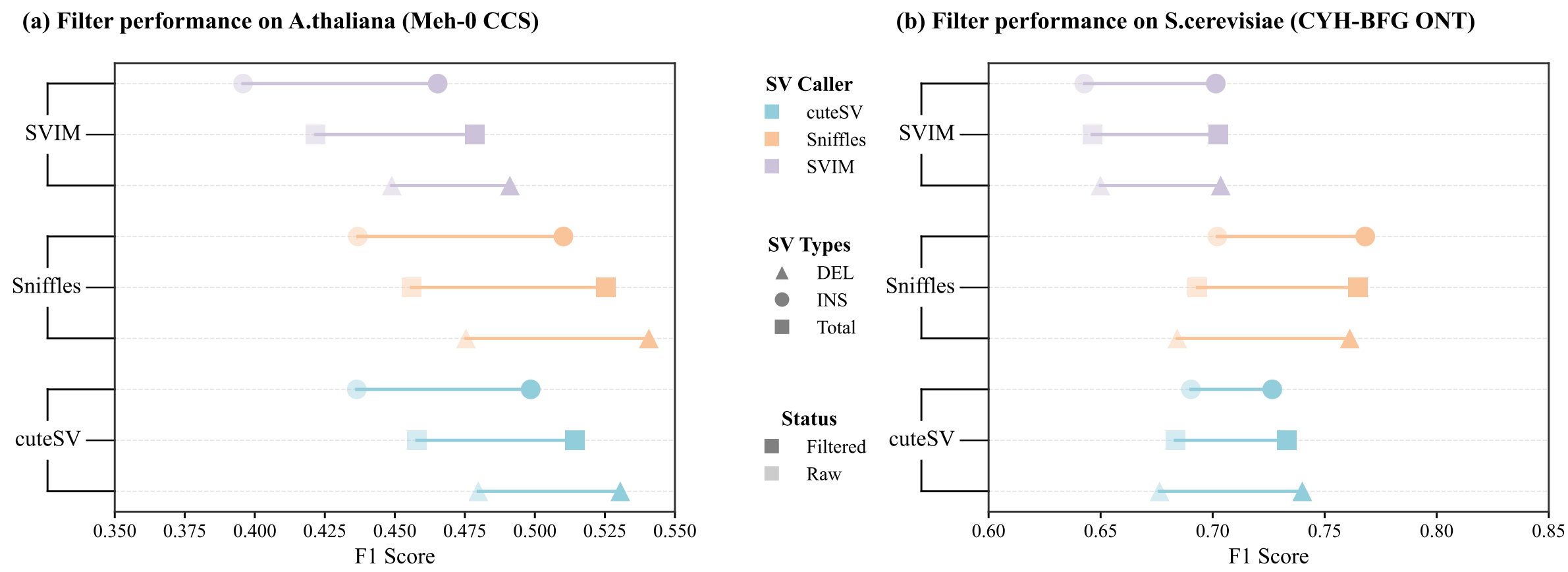

Figure 7: Performance gain analysis across independent plant and fungal samples. Detailed precision-recall metrics for the *A. thaliana* Meh-0 CCS and *S. cerevisiae* CYH-BFG ONT datasets. Panels compare the filtering efficacy across diverse platforms, illustrating the model's ability to transcend species-specific genomic patterns and effectively segregate heritable structural variants from stochastic noise.

6. Supplementary Tables

6.1. Mendelian discordance rate of SV filter

Table 14: Mendelian inheritance consistency analysis on the HG002 trio dataset.

SV	STATE	svim			sniffles			cutesv		
		Total	NotIn-Parents	MDR (%)	Total	NotIn-Parents	MDR (%)	Total	NotIn-Parents	MDR (%)
DEL	raw	13,928	1,450	10.41	13,356	1,320	9.88	10,460	1,280	12.24
	filter	12,311	980	7.96	13,036	980	7.52	9,766	900	9.22
INS	raw	13,545	1,550	11.44	12,205	1,380	11.31	9,571	1,340	14.00
	filter	11,885	1,150	9.67	11,976	1,100	9.18	8,897	970	10.91
Total	raw	27,473	3,000	10.92	25,561	2,700	10.56	20,031	2,620	13.08
	filter	24,196	2,130	8.80	25,012	2,080	8.32	18,663	1,870	10.02

6.2. Performance of different back-bone architectures.

Table 15: Mendelian inheritance consistency analysis on the HG005 trio dataset.

SV	STATE	svim			sniffles			cutesv		
		Total	NotIn-Parents	MDR (%)	Total	NotIn-Parents	MDR (%)	Total	NotIn-Parents	MDR (%)
DEL	raw	15,974	1,800	11.27	14,023	1,450	10.34	11,244	1,420	12.63
	filter	14,088	1,120	7.95	13,645	1,080	7.92	10,778	1,020	9.46
INS	raw	16,027	2,000	12.48	12,641	1,580	12.50	10,341	1,550	14.98
	filter	13,898	1,300	9.36	12,219	1,220	9.98	9,922	1,080	10.89
Total	raw	32,001	3,800	11.87	26,664	3,030	11.36	21,585	2,970	13.76
	filter	27,986	2,420	8.65	25,864	2,300	8.89	20,700	2,100	10.14

Table 16: Performance comparison of Semantic and Syntactic modalities across different backbone architectures.

Modality	Backbone	Precision	Recall	F1-score
Semantic (base)	ResNet18	0.9110	0.8140	0.8598
	ResNet50	**0.9227**	**0.8377**	**0.8781**
	ResNet101	0.9159	0.8184	0.8644
	MobileNetV2	0.9051	0.8112	0.8555
Syntactic (CIGAR)	ResNet18	0.9321	0.9176	0.9248
	ResNet50	0.9342	0.9223	0.9282
	ResNet101	0.9340	0.9255	0.9298
	MobileNetV2	**0.9476**	**0.9281**	**0.9378**

To investigate the influence of backbone architectures on modality-specific feature representation, we evaluated MobileNetV2 alongside several ResNet variants. As demonstrated in Table. 16, a distinct divergence in performance was observed between the two modalities, motivating our asymmetric dual-stream design:

- **Syntactic Modality and MobileNetV2:** Analysis reveals that the syntactic modality, derived from CIGAR strings, exhibits highly structured and noise-robust patterns. In this modality, the lightweight MobileNetV2 achieved the peak performance with an F_1-score of 0.9378 and precision of 0.9476. This indicates that for alignment-based features with clear topological signals, a lightweight architecture is sufficient to capture essential information while maintaining high computational efficiency.

- **Semantic Modality and ResNet-50:** Conversely, the semantic modality based on raw nucleotide sequences shows greater inherent ambiguity, with recall rates generally falling

below 0.83. In this context, ResNet-50 significantly outperformed both shallower and deeper variants, attaining the highest F_1-score of 0.8781. This suggests that the residual structure of ResNet-50 is better equipped to capture long-range dependencies and complex biological semantics within noisy genomic regions.

Owing to the intrinsic complexity and heterogeneous nature of genomic sequences, the semantic modality faces greater convergence challenges than structured CIGAR syntax signals in standalone training, resulting in a lower independent F_1-score. This outcome directly highlights the compelling need for multimodal integration. While the high-dimensional biological semantics derived from ResNet-50 offer limited individual discriminative power, they act as a vital regularizer when integrated into the syntactic flow.

Consequently, this asymmetric configuration justifies the employment of MobileNetV2 in the syntactic branch for efficiency and ResNet-50 in the semantic branch for enhanced regional modeling. By leveraging cross-modal synergy, the decoupled streams deliver crucial error-correction cues, successfully mitigating localized alignment artifacts and achieving optimal feature complementarity during the SV refinement process.
